# Increasing Interspecific Difference of Alpine Herb Phenology on the Eastern Qinghai-Tibet Plateau

**DOI:** 10.3389/fpls.2022.844971

**Published:** 2022-03-22

**Authors:** Shuai An, Xiaoqiu Chen, Miaogen Shen, Xiaoyang Zhang, Weiguang Lang, Guohua Liu

**Affiliations:** ^1^College of Applied Arts and Science, Beijing Union University, Beijing, China; ^2^Laboratory for Earth Surface Processes of the Ministry of Education, College of Urban and Environmental Sciences, Peking University, Beijing, China; ^3^State Key Laboratory of Earth Surface Processes and Resource Ecology, Faculty of Geographical Science, Beijing Normal University, Beijing, China; ^4^Geospatial Sciences Center of Excellence, Department of Geography and Geospatial Sciences, South Dakota State University, Brookings, SD, United States; ^5^Jiangsu Key Laboratory of Agricultural Meteorology, Nanjing University of Information Science and Technology, Nanjing, China

**Keywords:** alpine grassland, herbaceous plants, green-up and brown-off date, interspecific difference, climate change, sensitivities, long-term adaption

## Abstract

The phenology of alpine grassland on the Qinghai–Tibet Plateau (QTP) is critical to regional climate change through climate–vegetation feedback. Although many studies have examined QTP vegetation dynamics and their climate sensitivities, the interspecific difference in the phenology response to climate change between alpine species is poorly understood. Here, we used a 30-year (1989–2018) record of *in situ* phenological observation for five typical alpine herbs (*Elymus nutans*, *Kobresia pygmaea*, *Plantago asiatica*, *Puccinellia tenuiflora*, and *Scirpus distigmaticus*) and associated climatic records at Henan Station in the eastern QTP to examine the species-level difference in spring and autumn phenology and then quantify their climate sensitivities. Our results show that with significantly warming, the green-up dates of herbs were insignificantly shifted, while the brown-off dates in four out of the five herbs were significantly delayed. Meanwhile, the interspecific difference in brown-off dates significantly increased at a rate of 0.62 days/annual from 1989 to 2016, which was three times larger than that in green-up dates (0.20 days/annual). These diverse rates were attributed to the different climate controls on spring and autumn phenology. In particular, green-up dates in most herbs were sensitive to mean surface temperature, while brown-off dates were sensitive to the night surface temperature. Furthermore, brown-off dates are less sensitive to the warming in high ecological niche (with higher herb height and aboveground biomass) herbs than low niche herbs (with lower herb height and aboveground biomass). The increased phenology interspecific difference highlights the complex responses of herbs to future climate change even under the same alpine environment and indicates a potential alternation in the plants community of alpine QTP, which may further influence the regional climate–vegetation feedback.

## Introduction

Plant phenology is a sensitive indicator of climate change (Menzel, 2002). With climate influences on the seasonal vegetation growth, phenology also has important impacts on ecosystem production, land–atmosphere interactions, as well as the biogeochemical cycle, structure, and function (Piao et al., 2019). Known as the highest Plateau in the world, the Qinghai–Tibet Plateau (QTP) has been experiencing dramatic warming since the 1980s, where the warming rate is almost two times as the global average (Chen et al., 2013). As a key ecosystem indicator, the phenology of alpine grassland and its response to climate change on the QTP have received extensive attention from the academic community (Shen et al., 2015). Previous studies have been intensively using satellite remote sensing data to examine the land surface phenology of alpine grassland at the pixel (community) scale (Zhang et al., 2013; Shen et al., 2016; An et al., 2020; Li et al., 2021). However, it cannot provide information on the species-level changes and the interspecific difference in phenology as they are resolved in pixels, which largely limits our ability to accurately assess the climate change impact on the alpine herb dynamics.

Based on long-term *in situ* observations, some studies have examined the interspecific difference of herbs phenology for grassland and forest ecosystems, including temperature grasslands, alpine grasslands, and temperate deciduous forests. For example, during the past three decades, at the stations in Inner Mongol of China, the green-up/brown-off dates of temperate herb species in the same station showed diverse linear trends (Huang et al., 2019; Wang et al., 2019). With the same warming amplitude in the past two decades at Haibei Station in the northeastern QTP, eight herb species green-up dates showed divergent linear trends, including advances and delays of varying magnitudes (Chen et al., 2015a; Sun et al., 2020), as well as for the brown-off dates (Sun et al., 2020). A similar phenomenon was also found in herbs of temperate deciduous forest in the United States in the past two decades (Augspurger and Zaya, 2020), in which herb species differed greatly in the degree or direction of spring/autumn phenological variations. It has been widely recognized that abiotic factors could stress the plant phenology (Cleland et al., 2007; Pau et al., 2011). However, the mechanisms behind for the divergent phenological responses of different herb species to abiotic factors, such as temperature, precipitation, sunshine duration, and other climate factors at the same station have still not been clearly illustrated.

The *in situ* controlled experiment in the Haibei Station demonstrated that nutrient addition increased the competitive advantage of *Gramineae* against *Cyperaceae*, so that the green-up date was advanced for *Cyperaceae* plants in a competitively disadvantaged environment advanced while delayed for *Gramineae* (Ye et al., 2014). With moisture addition, the brown-off date for *Elymus nutans* and *Scirpus distigmaticus* was significantly delayed but it was advanced for forbs (Ye et al., 2014). It means that the phenology sensitivities of different herbs to the change of environmental factors are various. Because the experiment (Ye et al., 2014) was only carried out for 3 years, it is hard to explore the mechanism of climate controls on the interspecific difference of herb phenology on the QTP for a long term. Regarding the response of herb phenology to climate change, the green-up/brown-off dates of temperate herb species displayed various sensitivities to thermal–moisture factors in the Inner Mongol of China (Huang et al., 2019; Ren et al., 2022). Such diverse phenology responses of different herb species at the same stations were also found in alpine grasslands on the QTP in China (Suonan et al., 2019; Wang et al., 2020). Besides, other *in situ* controlled experiments, such as manipulative experimental warming and rainfall interception also indicated that spring/autumn phenology of different herb species have various sensitivities (Suonan et al., 2017; Ganjurjav et al., 2020; Luo et al., 2021).

Based on the *in situ* phenology data of five typical herbs, height, aboveground biomass, and the parallel climate data from 1989 to 2018 in Henan Station at the eastern QTP, we aim to answer the following questions: (1) What is the interspecific difference of alpine herb spring and autumn phenology variation and its climate response in the past three decades at the eastern QTP? (2) What is the interspecific difference of climate sensitivities for alpine herb phenology in long term? (3) What kinds of climate adaption strategies would be adopted by the alpine herb phenology? The result could provide evidence for divergent climate change responses of alpine grassland herbs on the Qinghai–Tibet Plateau under global warming.

## Materials and Methods

### Study Area

Henan Station is located in the eastern Qinghai–Tibet Plateau in China (Supplementary Figure 1) with a geographical coordinate of 34°44′N and 101°36′E and an elevation of 3,500 m. Its climate belongs to typical plateau continental climates with an annual mean temperature of −0.2°C and annual precipitation of 568.1 mm (Chen et al., 2015a). The vegetation type in this station is classified as alpine meadow, where dominant species consists of *E. nutans*, *Festuca ovina*, *Kobresia humilis*, *Kobresia pygmaea*, *Poa crymophila*, *Puccinellia tenuiflora*, and *S. distigmaticus* (Du et al., 2015).

### Phenology and Climate Data

Phenology and climate data sets were obtained from the China Meteorological Administration^1^. The phenology data include green-up/brown-off dates of five typical herbs, namely, *E. nutans*, *K. pygmaea*, *Plantago asiatica*, *P. tenuiflora*, and *S. distigmaticus* (Supplementary Table 1). The observation station is a fenced area of 100 m × 100 m. In the field observation, 10 fixed individuals were chosen for each herb species and the phenological observation was carried out every 2 days by professionals according to uniform observation criteria (China Meteorological Administration, 1993). Specifically, the green-up date was identified as the date when 50% (5 of 10) of the herb individuals display green leaves and grow up higher than 1 cm above the ground in spring, and the brown-off date was identified as the date when 50% (5 out of 10) of herb individuals turn two-thirds of their aboveground body into yellow in autumn (China Meteorological Administration, 1993; Chen et al., 2015a; Sun et al., 2018). The growing season length was measured as the difference between the green-up and the brown-off dates. Standard deviations of green-up/brown-off dates of the five herbs in each year were calculated to measure the interspecific difference of the phenology. The climatic variables include daily minimum/maximum/mean ground surface temperature (T*_*gmin*_*, T*_*gmax*_*, T*_*gmean*_*), daily minimum/maximum/mean air temperature (T*_*min*_*, T*_*max*_*, T*_*mean*_*), daily precipitation (Pre), daily sunshine duration (Ssd), daily minimum/mean relative humidity (H*_*min*_*, H*_*mean*_*), daily mean/maximum wind speed (W*_*mean*_*, W*_*max*_*), and daily evaporation (Eva) from 1989 to 2018.

### Herb Height and Aboveground Biomass Data Sets

Herb height was observed and recorded every 10 days in the growing season of a year and aboveground biomass (AGB) in fresh weight was harvested and measured at the end of May, June, July, and August by the professionals (China Meteorological Administration, 1993). The data sets were also obtained from the China Meteorological Administration (see text footnote 1). The annual mean value of maximum herb height and AGB were computed to represent relative ecological niches for each herb species.

### Statistical Analyses of Long-Term Herb Phenology and Its Response to Climatic Variables

The Theil–Sen estimator and Mann–Kendall trend test (Chen et al., 2015b) were used to fit and test the linear trends of the herb green-up/brown-off date, growing season length, and climatic factors. These non-parametric statistics have been proved to be appropriate for detecting the linear trend, especially when time series are subject to spatial or temporal autocorrelation. The Pearson correlation analysis was used to calculate the correlation coefficients between herb green-up/brown-off dates and monthly mean climatic variables within 3 months before the multiyear mean green-up/brown-off date.

The linear regression models that simulate plant phenology based on climatic factors in optimum period assume that there is an optimum period length for the impact of climatic factors to plant phenology (Matsumoto et al., 2003; Chen and Xu, 2012). In our study, the optimum period length is determined by the maximum absolute value of the coefficient of Pearson correlation between the average or cumulative value of a climate factor in a time window (1 day) preceding the phenological dates and green-up/brown-off date. Stepwise regression models were used to simulate herb phenology based on optimum period length climatic factor time series and the regression coefficient with each independent variable was applied to explain the sensitivities of herb green-up/brown-off dates to the variation of climate factors. Details of the method could be found in An et al.’s (2020) study. All the statistical analyses are realized in the MATLAB 2016a programming environment.

## Results

### Linear Trends of Different Climatic Indicators

**FIGURE 1:**
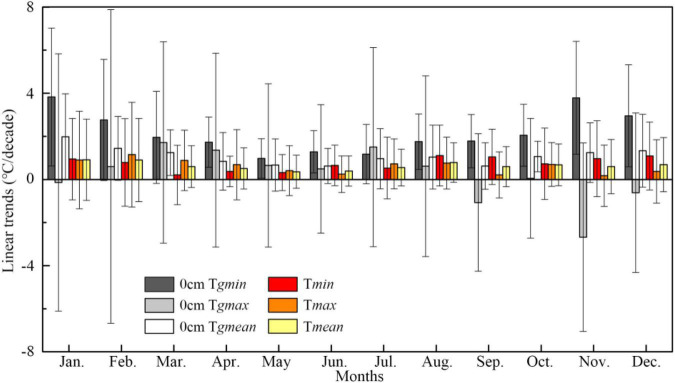
Linear trends and standard errors of temperature in each month from 1989 to 2018 in Henan Station. The horizontal axis is the month in a year, and the vertical axis is the slope of the linear trend. The black/gray/white and red/orange/yellow bars represent minimum/maximum/mean daily 0 cm ground temperature (T*_*gmin*_*, T*_*gmax*_*, and T*_*gmean*_*) and minimum/maximum/mean air temperature (T*_*min*_*, T*_*max*_*, and T*_*mean*_*), respectively. Details of the statistics of the linear trends could be found in Supplementary Table 2.

**FIGURE 2:**
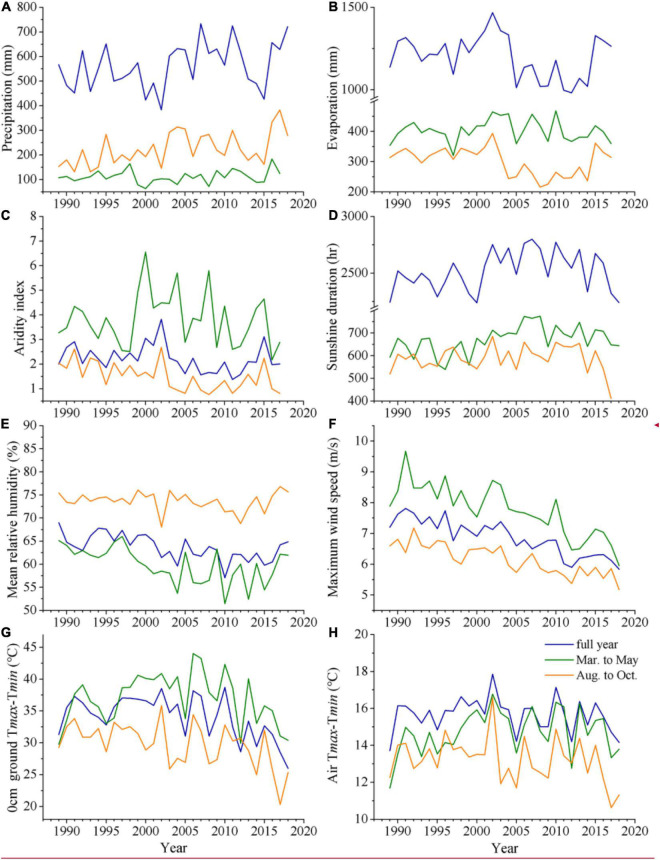
Variations in accumulation precipitation **(A)**, evaporation **(B)**, aridity index (defined as evaporation/precipitation) **(C)**, sunshine duration **(D)**, mean relative humidity **(E)**, maximum wind speed **(F)**, 0 cm ground temperature difference between day and night **(G)** and air temperature difference between day and night **(H)** in March–May, August–October, and full year from 1989 to 2018.

The mean daily minimum ground temperature (T*_*gmin*_*) showed significant warmings in all months (*P* < 0.001) and the warming rates were larger than those of T*_*gmean*_*, T*_*min*_*, T*_*max*_*, and T*_*mean*_* from 1989 to 2018 (Figure 1 and Supplementary Table 2). Moreover, T*_*gmin*_* warming rates in the autumn and winter months were higher than that in the spring and summer months. Meanwhile, significantly increased trends were found in August–October precipitation (3.91 mm/a, *P* = 0.0067) (Figure 2A) and March–May daily sun duration (2.86 h/a, *P* = 0.0145) (Figure 2D). In contrast, the significantly decreased trends appeared in evaporation (−2.54 mm/a, *P* = 0.0356) (Figure 2B); aridity index in August–October (−0.04/a, *P* = 0.0017), and full year (−0.02/a, *P* = 0.0409) (Figure 2C); mean relative humidity in March—May (−0.28%/a, *P* = 0.0024) and full year (−0.21%/a, *P* = 0.0012) (Figure 2E); daily maximum wind speed in March–May (−0.08 m/s/a, *P* < 0.001), August–October (−0.05 m/s/a, *P* < 0.001), and full year (−0.06 m/s/a, *P* < 0.001) (Figure 2F); as well as surface diurnal range in August–October (−0.19°C/a, *P* = 0.0054), and full year (−0.21°C/a, *P* = 0.0204) (Figure 2G and Supplementary Table 3).

### Linear Trends of Phenology in Five Herb Species

**FIGURE 3:**
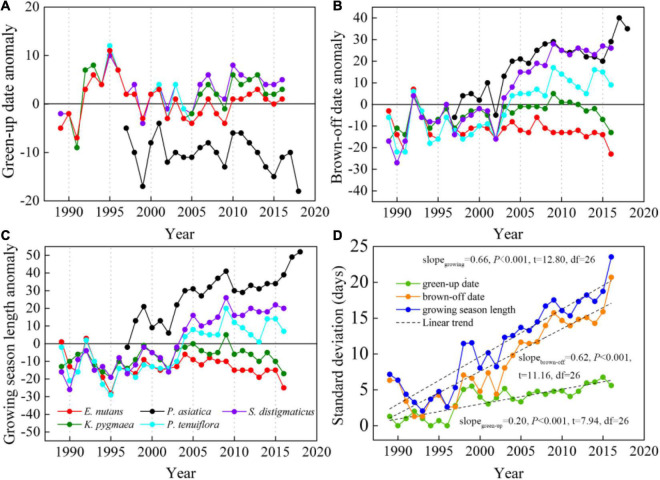
Anomaly of herb green-up date **(A)**, brown-off date **(B)**, growing season length **(C),** and the interspecies standard deviations **(D)** in Henan Station from 1989 to 2018. Five different color legends represent different herbs, respectively. Slope_*green*–up_, slope_*brown*–off_, and slope_*growing*_ in **(D)** represent the linear trend slopes of standard deviations of the five herbs (*Elymus nutans*, *Kobresia pygmaea*, *Plantago asiatica*, *Puccinellia tenuiflora*, and *Scirpus distigmaticus*) green-up dates, brown-off dates, and growing season lengths, respectively.

The linear trends of phenology from 1989 to 2018 differed greatly among the five herb species (Supplementary Tables 4–6). There was no significant trend in herb green-up dates (Figure 3A). In contrast, brown-off date was insignificantly advanced for *E. nutans* (−0.20 days/annual, *P* = 0.07) but significantly delayed for the other four herbs (*P* < 0.02 or *P* < 0.001) with a rate of 0.29 days/annual for *K. pygmaea*, 1.23 days/annual for *P. tenuiflora*, 1.58 days/annual for *P. asiatica*, and 1.84 days/annual for *S. distigmaticus* (Figure 3B). Thus, the growing season was significantly prolonged (*P* < 0.001) for *P. asiatica*, *P. tenuiflora*, and. *distigmaticus* with a rate of 1.75, 1.25, and 1.67 days/annual, respectively (Figure 3C). Furthermore, standard deviations among the five herb species in green-up, brown-off dates, and growing season length were all significantly increased from 1989 to 2016 (Figure 3D). Linear trend slopes of interspecific standard deviations in brown-off date and growing season length were 0.62 days/annual and 0.66 days/annual (*P* < 0.001), which were three times larger than that for herbs green-up dates (0.20 days/annual, *P* < 0.001) (Figure 3D).

### Correlation Between Herb Phenology and Climatic Variables

**FIGURE 4:**
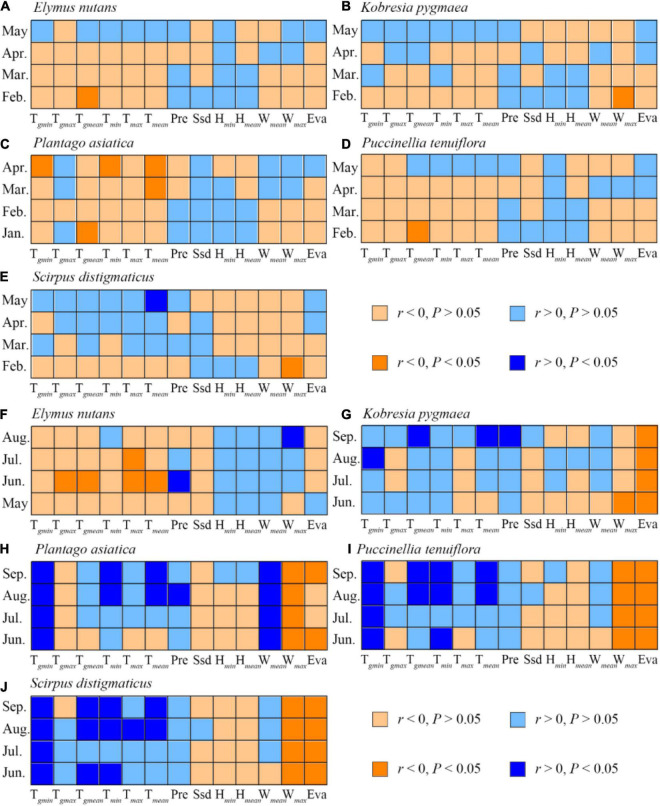
Pearson correlation coefficients (*r*) between five herbs green-up/brown-off dates and climatic factors mean/accumulation values in annual mean green-up/brown-off date month and its pre 3 months. Panels **(A–E)** are green-up dates for *Elymus nutans*, *Kobresia pygmaea*, *Plantago asiatica*, *Puccinellia tenuiflora*, and *Scirpus distigmaticus*, respectively. Panels **(F–J)** are brown-off dates for them. T*_*gmin*_*, T*_*gmax*_*, T*_*gmean*_*, T*_*min*_*, T*_*max*_*, T*_*mean*_*, Pre, Ssd, H*_*min*_*, H*_*mean*_*, W*_*mean*_*, W*_*max*_*, and Eva represent daily minimum/maximum/mean 0 cm ground temperature, daily minimum/maximum/mean air temperature, daily precipitation, daily sunshine duration, daily minimum/mean relative humidity, daily mean/maximum wind speed, and daily evaporation, respectively. Details of the statistics of the Pearson correlation coefficients could be found in Supplementary Table 7.

Greenup/brown-off dates in different species were correlated to climatic variables differently. A significantly positive correlation was found between *E. nutans* and *K. pygmaea* green-up dates and the February T*_*gmean*_* and February W*_*max*_*, respectively; between *P. asiatica* green-up date and January, May, and April mean temperature (T*_*gmin*_*, T*_*gmean*_*, T*_*min*_*, T*_*mean*_*), respectively; as well as between *P. tenuiflora* green-up date and February T*_*gmean*_*. However, *S. distigmaticus* green-up date was significantly negatively correlated with May T*_*min*_* but positively correlated with February W*_*max*_* (Figures 4A–E and Supplementary Table 7).

*Elymus nutans* brown-off date was significantly and negatively correlated with the June mean temperature (T*_*gmax*_*, T*_*gmean*_*, T*_*max*_*, T*_*mean*_*) and positively with June accumulation Pre and August mean W*_*max*_*. *Kobresia pygmaea* brown-off date was significantly and positively correlated with the August mean T*_*gmin*_*, September mean temperature (T*_*gmean*_*, T*_*mean*_*) and accumulation Pre, and negatively correlated with May–June mean W*_*max*_*, June–September accumulation Eva. Both *P. asiatica*, *P. tenuiflora*, and *S. distigmaticus* brown-off dates were all significantly positively correlated with the August–September temperature (T*_*gmin*_*, T*_*gmean*_*, T*_*min*_*, T*_*mean*_*) and negatively with June–September mean W*_*max*_*, and accumulation Eva. In addition, *P. asiatica* brown-off date was also positively correlated with August accumulation Pre and June–September mean W*_*mean*_* (Figures 4F–J and Supplementary Table 7).

### Sensitivities of Herb Green-Up/Brown-Off Dates to Climatic Variables

Stepwise regression models selected the most sensitive climatic variables in controlling herb phenology (Supplementary Tables 8, 9). (1) *Elymus nutans* green-up date was sensitive to T*_*gmean*_* and T*_*max*_* with an advance rate of 1.72 days/°C and delay rate of 1.22 days/°C, respectively, while *E. nutans* brown-off date was sensitive to T*_*max*_* with an advance rate of 5.1 days/°C. (2) *Kobresia pygmaea* green-up date was advanced by 0.51 days with 1% H*_*mean*_* increase, while *K. pygmaea* brown-off date was advanced by 0.04 days with 10 mm evaporation increase. (3) *Plantago asiatica* green-up date was controlled by T*_*gmean*_* and Ssd with an advance rate of 2.49 days/°C and 0.09 days/h, respectively. *Plantago asiatica* brown-off date was sensitive to T*_*gmin*_* and W*_*max*_*. (4) *Puccinellia tenuiflora* green-up date was controlled by T*_*gmean*_* and Eva, which was advanced for 1.20 days/°C and delayed for 0.09 days/mm, respectively. *Puccinellia tenuiflora* brown-off was sensitive to T*_*gmin*_* and Eva. (5) While *S. distigmaticus* green-up was modeled by T*_*min*_* and H*_*mean*_*, *S. distigmaticus* brown-off date was sensitive to T*_*gmin*_* and H*_*mean*_*. For the three herbs of *S. distigmaticus*, *P. asiatica*, and *P. tenuiflora*, brown-off date was delayed by 9.68, 6.66, and 4.54 days, respectively, with 1°C increase of T*_*gmin*_*, which was also negatively sensitive to W*_*max*_*, H*_*mean*_*, and Eva.

### Height and Aboveground Biomass Difference Among the Alpine Herbs in Henan Station

**FIGURE 5:**
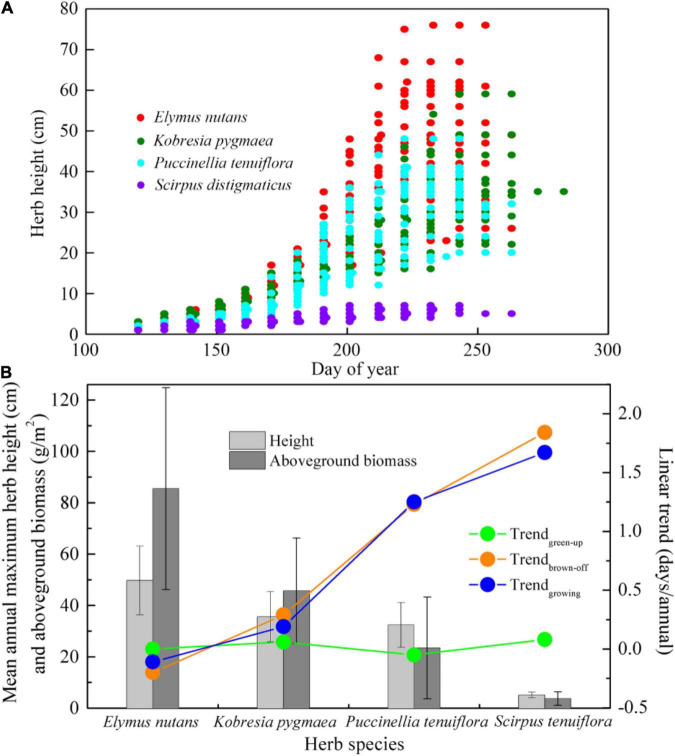
**(A)** Distribution of herb heights of four typical alpine herb species (*Elymus nutans*, *Kobresia pygmaea*, *Puccinellia tenuiflora*, and *Scirpus distigmaticus*) during the growing season from 1994 to 2012 in Henan Station. **(B)** Comparison among different herb species in multi-year mean annual maximum herb height, aboveground biomass, and linear trends of green-up, brown-off date, and growing season length from 1989 to 2018 in Henan Station. Trend_*green–up*_, Trend_*brown–off*_, and Trend_*growing*_ represent the linear trends of the four herbs green-up dates, brown-off dates, and growing season lengths, respectively.

The heights distribution of four typical alpine herbs (*E. nutans*, *K. pygmaea*, *P. tenuiflora*, and *S. distigmaticus*) in the growing season from 1994 to 2012 are presented in Figure 5A. Specifically, *Elymus nutans* was higher than other herbs. The multiyear mean of annual maximum herb height was 49.7 cm (Figure 5B) and the highest record was close to 80 cm (Figure 5B), followed by *K. pygmaea* (35.6 cm, 59 cm). The maximum height (32.4 cm, 48 cm) of *P. tenuiflora* was slightly lower than *K. pygmaea*. However, the height of *S. distigmaticus* was the lowest, with the mean value of 5.1cm and the highest record less than 10 cm. The order of multiyear mean of annual maximum herb aboveground biomass in fresh weight showed a similar pattern (Figure 5B), namely, *E. nutans* (85.5 g/m^2^), *K. pygmaea* (45.7 g/m^2^), *P. tenuiflora* (23.4 g/m^2^), and *S. distigmaticus* (3.7 g/m^2^).

## Discussion

### Increased Interspecific Difference of Herb Phenology Under Long-Term Warming

Our study found that the alpine herbs green-up dates did not show significant trends at Henan Station on the eastern QTP from 1989 to 2018, whereas most dominant herb brown-off dates were significantly delayed, which is similar to the results of delayed autumn land surface phenology in the eastern QTP base on AVHRR and MODIS satellite remote sensing data analyses (Che et al., 2014; Zu et al., 2018; Li et al., 2019). This indicates a high degree of consistency between species-level and community-level (pixel-level) phenology variation at Henan Station. Consequently, the herbs growing season was mainly extended (Sun et al., 2020), which could further impact the grassland ecosystem structure and function (Fridley et al., 2016; Leblans et al., 2017). The delayed brown-off dates in most herbs are likely associated with climate conditions. These include (1) that the warming amplitude in autumn months was higher than that in spring months, especially at night surface temperature, and (2) the surface/air temperature difference between day and night in autumn months (Figures 2G,H) was dramatically decreased and the change rate was also higher than that in spring months, indicating an improved heat condition at night in Henan Station. The warming climate could result in a better growth environment for the herb development. However, different herb species showed distinctive trajectories of the brown-off dates (Figure 3B), where the interspecific difference increase rate was even three times larger than that of the green-up date which also showed obvious interspecies difference (Figure 3D). This suggests that the response of interspecies difference of herbs phenology to the climate in autumn is more drastic than spring phenology stages.

The herb green-up/brown-off dates were mainly driven by ground heat conditions in Henan Station (Li et al., 2011). They were particularly correlated to T*_*gmean*_* and T*_*gmin*_* based on Pearson correlation and stepwise regression analyses, showing the higher the T*_*gmean*_* and T*_*gmin*_*, the earlier the herbs green, the later the herbs senesce. This pattern is attributed to the low temperature that would enhance herb stress and immensely damage the leaf membrane system (Dong et al., 2020), which in turn affects the growth and development of the herbs (Chen et al., 2020) and *vice versa*.

### Relationships Between Herb Height/Aboveground Biomass and Climate Sensitivity of Phenology

The sensitivity of herb phenology, especially brown-off date, to the surface temperature differed greatly among herb species. In particular, *S. distigmaticus* senescence is extremely sensitive to night surface temperature (9.68 d/°C), which resulted in the highest brown-off date delay rate (1.84 days/annual). Compared to other herbs, *S. distigmaticus* height (Figures 5A,B) and aboveground biomass (Figure 5B) are both the lowest, which indicates that it is in a weak competitive position in the grassland community for light and water (Pau et al., 2011). To fight for more ecological resources, *S. distigmaticus* may have tried its best to delay the senescence date and extended the growing season length under suitable hydrothermal conditions, resulting in the highest delayed rate for autumn phenology (Figure 5B). As a wide ecological amplitude widespread weed, *P. asiatica* has greater adaptability to cope with the climate change, whose T*_*gmin*_* sensitivity of green-up date and advanced rate are the highest (−2.49 d/°C, −0.19 days/annual) and T*_*gmin*_* sensitivity of brown-off date and delayed rate followed *S. distigmaticus* (6.66 d/°C, 1.58 days/annual). In contrast, *P. tenuiflora* height and ground biomass are higher (Figure 5), which means stronger interspecific competitiveness. Thus, its T*_*gmin*_* sensitivity and delayed rate (4.54 d/°C, 1.17 days/annual, Figure 5) are lower.

Although herbs phenology at Henan Station is mainly controlled by temperature, the extreme drought still could accelerate the herb senescence. For example, the severe drought in 2002 caused a higher aridity index (Figure 2C), which intensified herb senescence and advanced the brown-off dates (Figure 2B). Usually, drought severity depends on the interaction of precipitation and evaporation. The latter depends on relative humidity, wind speed, and vegetation coverage. During the past three decades, the daily maximum wind speed significantly decreased, which could also reduce the evaporation. The sensitivities of herb phenology to relative humidity, wind speed, and evaporation make the phenological response more unintelligible. For example, *S. distigmaticus* green-up and brown-off dates are both negatively sensitive to relative humidity. *P. asiatica* and *P. tenuiflora* brown-off dates are both sensitive to daily mean/maximum wind speed, while they show the opposite effect. Because of the complex interaction mechanism of these climate factors for moisture (An et al., 2020), it is still hard to quantify the drought impact of herb phenology.

### Diverse Warming Adaption Strategies of Alpine Herbs

Differences in plant phenology are the embodiment of changes in plant life history. The sensitivity of each herb to the selected climatic factors varies greatly, which indicates different strategies for the herbs to adapt to warming in alpine environments. As a constructive species of alpine grassland in Henan Station, *K. pygmaea* is distributed in the high mountain area at an altitude of 3,100 to 5,600 m (Flora of China Editorial Committee of Chinese Academy of Sciences, 2010), and has stronger competitiveness. Protein level analysis shows that *K. pygmaea* has adapted to the high mountain ecological environment, including higher temperature, intense light, and ultraviolet radiation in the day and lower temperature in the nights (Li et al., 2020) and belongs to cold-resistant mesophytes. To avoid freezing damage, the sensitivity of vegetation phenology to temperature also decreases (Wang et al., 2014). Thus, *K. pygmaea* spring and autumn phenology are both less sensitive to warming but sensitive to humidity and evaporation, respectively. The greater advance of green-up date and significant delay of brown-off date of *P. asiatica* (Li, 2015; Gao, 2018) indicate that the weedy herbs are more ecologically adaptable to the environment change due to their wider ecological amplitude (Chen et al., 2015b). Moreover, disparate phenology responses have been demonstrated (Julitta et al., 2014; Huang et al., 2020; Ren et al., 2022) by process-based models (Chen et al., 2015a) for grass and forbs or native and exotic species (Prevéy et al., 2014; Lang et al., 2019). Thus, forbs or exotic species phenology seems more sensitive to warming, which could be an effective indicator for the warming. *Elymus nutans* spring and autumn phenological events are both relatively stable over the years and are sensitive to daily maximum air temperature. *Elymus nutans* belongs to xerophyte and is extremely frost and drought resistant; therefore, its phenology is not sensitive to night warming and moisture variation. In a word, different herb species adopt their unique strategies to adapt climate change, which depends on their ecological niche, ecotype, and ecological amplitude. It can be inferred that with the increase of interspecies difference in the response of phenology to the global warming, the herbs growing season length interspecies difference at Henan Station will also change, which in turn will cause interspecies difference variation in the alpine grassland ecosystem biogeochemical cycle.

## Conclusion

With the intense warming during the past three decades, the interspecific difference of alpine herb phenology continued to increase and the change rate of autumn phenology is much larger than that of spring phenology. The sensitivities of herb green-up/brown-off dates to climate variations also show large interspecies differences being sensitive, which results in exhibiting different climate change adaptation strategies of different grass species. The five herb species adopted different strategies to adapt to climate change, which depends on their ecological niches, ecotypes, and ecological amplitudes. Brown-off dates of the high ecological niche species (with higher herb height and aboveground biomass) are less sensitive to climate warming, while brown-off dates of the low ecological niche herbs (with lower herb height and aboveground biomass) are more sensitive. Interspecific competition might be an important cause of different sensitivities of herb brown-off dates to climate variation. As climate changes, interspecific competition has accelerated the interspecific difference of herb phenology, especially autumn phenology, which may further remodel the alpine grassland ecosystem structure and function. The impacts of this series of chain reactions requires further research through long-term ground control experiments.

## Data Availability Statement

The original contributions presented in the study are included in the article/Supplementary material, further inquiries can be directed to the corresponding author/s.

## Author Contributions

SA designed the experiment, performed the experiment, analyzed all data, and wrote the first draft of the manuscript. XC, MS, XZ, WL, and GL provided some comments and suggestions for the manuscript. All authors contributed to the manuscript revision.

## Conflict of Interest

The authors declare that the research was conducted in the absence of any commercial or financial relationships that could be construed as a potential conflict of interest.

## Publisher’s Note

All claims expressed in this article are solely those of the authors and do not necessarily represent those of their affiliated organizations, or those of the publisher, the editors and the reviewers. Any product that may be evaluated in this article, or claim that may be made by its manufacturer, is not guaranteed or endorsed by the publisher.

## Footnotes

1
http://www.nmic.cn/
