# Supplementary material for: Increasing Interspecific Difference of Alpine Herb Phenology on the Eastern Qinghai-Tibet Plateau
Source: Front Plant Sci. 2022 Mar 22;13:844971. doi: 10.3389/fpls.2022.844971 (PMC8982063; doi:10.3389/fpls.2022.844971)
Supplement: Supplementary file 1 [file Data_Sheet_1.docx]

**SUPPLEMENTARY MATERIAL**


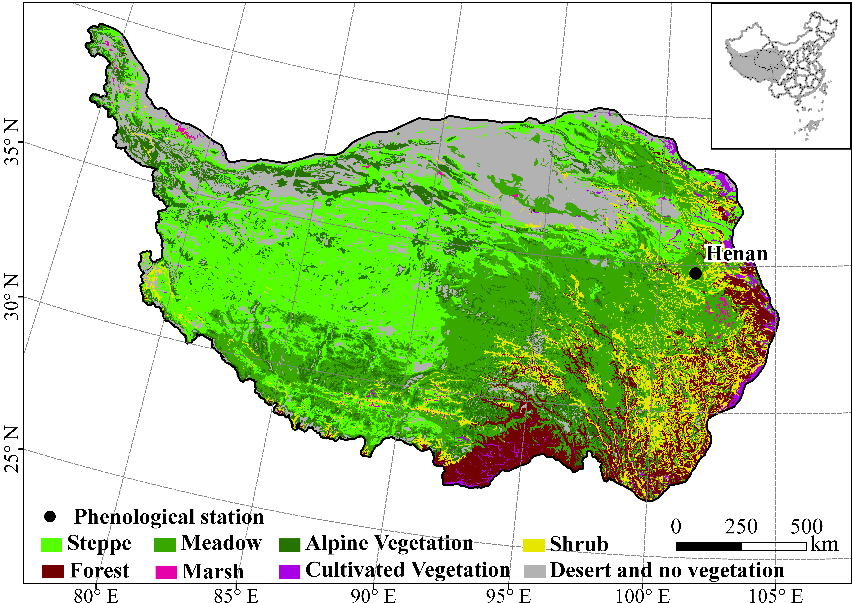


**Supplementary Figure 1.** Vegetation types and Henan Station location in the Qinghai-Tibet Plateau

**Supplementary Table 1** Basic information of herbs in Henan Station

| Species | Family | Group | Niche | Ecotype | Ecological amplitude |
| --- | --- | --- | --- | --- | --- |
| *E. nutans* | Gramineae | forage | dominant | xerophyte | narrow |
| *K*. *pygmaea* | Cyperaceae | forage | constructive | mesophyte | narrow |
| *P*. *asiatica* | Plantaginaceae | weed | accompanying | mesophyte | wide |
| *P*. *tenuiflora* | Gramineae | forage | dominant | xerophyte | wide |
| *S*. *distigmaticus* | Cyperaceae | forage | dominant | xero-mesophyte | narrow |

**E. nutans*, *K*. *pygmaea*, *P*. *asiatica*, *P*. *tenuiflora* and *S*. *distigmaticus* are the abbreviations of *Elymus nutans*, *Kobresia pygmaea*, *Plantago asiatica*, *Puccinellia tenuiflora* and *Scirpus distigmaticus*

**Supplementary Table 2** Test statistics for the linear trends of temperature in each month from 1989 to 2018 in Henan Station

| Temperature | Month | Liner trend  (d/a) | t-statistics | df | P-value | Standard error | Confidence interval  (%) |
| --- | --- | --- | --- | --- | --- | --- | --- |
| T*_gmin_* | 1 | 3.83 | 5.67 | 28 | 0.0000 | 3.20 | 99 |
| T*_gmin_* | 2 | 2.76 | 4.66 | 28 | 0.0001 | 2.81 | 99 |
| T*_gmin_* | 3 | 1.95 | 4.34 | 28 | 0.0002 | 2.13 | 95 |
| T*_gmin_* | 4 | 1.73 | 7.05 | 28 | 0.0000 | 1.16 | 99 |
| T*_gmin_* | 5 | 0.97 | 5.05 | 28 | 0.0000 | 0.91 | 99 |
| T*_gmin_* | 6 | 1.28 | 6.20 | 28 | 0.0000 | 0.98 | 99 |
| T*_gmin_* | 7 | 1.17 | 4.04 | 28 | 0.0004 | 1.38 | 99 |
| T*_gmin_* | 8 | 1.75 | 6.45 | 28 | 0.0000 | 1.29 | 99 |
| T*_gmin_* | 9 | 1.78 | 6.85 | 28 | 0.0000 | 1.23 | 99 |
| T*_gmin_* | 10 | 2.05 | 6.77 | 28 | 0.0000 | 1.44 | 99 |
| T*_gmin_* | 11 | 3.79 | 6.86 | 28 | 0.0000 | 2.62 | 99 |
| T*_gmin_* | 12 | 2.95 | 5.92 | 28 | 0.0000 | 2.36 | 99 |
| T*_gmax_* | 1 | -0.14 | -0.11 | 28 | 0.9136 | 5.97 | 5 |
| T*_gmax_* | 2 | 0.60 | 0.39 | 28 | 0.6981 | 7.28 | 30 |
| T*_gmax_* | 3 | 1.72 | 1.74 | 28 | 0.0927 | 4.67 | 90 |
| T*_gmax_* | 4 | 1.36 | 1.43 | 28 | 0.1626 | 4.50 | 80 |
| T*_gmax_* | 5 | 0.65 | 0.82 | 28 | 0.4214 | 3.79 | 55 |
| T*_gmax_* | 6 | 0.49 | 0.77 | 28 | 0.4458 | 2.98 | 55 |
| T*_gmax_* | 7 | 1.50 | 1.54 | 28 | 0.1348 | 4.62 | 85 |
| T*_gmax_* | 8 | 0.62 | 0.70 | 28 | 0.4914 | 4.20 | 50 |
| T*_gmax_* | 9 | -1.07 | -1.59 | 28 | 0.1234 | 3.19 | 85 |
| T*_gmax_* | 10 | 0.05 | 0.09 | 28 | 0.9284 | 2.78 | 5 |
| T*_gmax_* | 11 | -2.68 | -2.91 | 28 | 0.0071 | 4.37 | 99 |
| T*_gmax_* | 12 | -0.61 | -0.79 | 28 | 0.4374 | 3.70 | 55 |
| T*_gmean_* | 1 | 1.98 | 4.69 | 28 | 0.0001 | 2.00 | 99 |
| T*_gmean_* | 2 | 1.44 | 4.60 | 28 | 0.0001 | 1.49 | 99 |
| T*_gmean_* | 3 | 1.24 | 5.58 | 28 | 0.0000 | 1.06 | 99 |
| T*_gmean_* | 4 | 0.84 | 2.97 | 28 | 0.0061 | 1.34 | 99 |
| T*_gmean_* | 5 | 0.67 | 2.62 | 28 | 0.0139 | 1.21 | 95 |
| T*_gmean_* | 6 | 0.62 | 3.62 | 28 | 0.0011 | 0.82 | 99 |
| T*_gmean_* | 7 | 0.96 | 3.26 | 28 | 0.0030 | 1.40 | 99 |
| T*_gmean_* | 8 | 1.04 | 3.32 | 28 | 0.0025 | 1.48 | 99 |
| T*_gmean_* | 9 | 0.62 | 2.74 | 28 | 0.0107 | 1.08 | 95 |
| T*_gmean_* | 10 | 1.06 | 7.09 | 28 | 0.0000 | 0.71 | 99 |
| T*_gmean_* | 11 | 1.24 | 4.25 | 28 | 0.0002 | 1.39 | 99 |
| T*_gmean_* | 12 | 1.33 | 3.74 | 28 | 0.0008 | 1.69 | 99 |
| T*_min_*, | 1 | 0.94 | 2.36 | 28 | 0.0255 | 1.89 | 95 |
| T*_min_* | 2 | 0.79 | 1.83 | 28 | 0.0772 | 2.04 | 90 |
| T*_min_* | 3 | 0.21 | 0.72 | 28 | 0.4798 | 1.38 | 50 |
| T*_min_* | 4 | 0.37 | 2.50 | 28 | 0.0187 | 0.71 | 95 |
| T*_min_* | 5 | 0.31 | 1.79 | 28 | 0.0848 | 0.84 | 90 |
| T*_min_* | 6 | 0.65 | 3.27 | 28 | 0.0028 | 0.94 | 99 |
| T*_min_* | 7 | 0.53 | 1.75 | 28 | 0.0903 | 1.43 | 90 |
| T*_min_* | 8 | 1.11 | 3.73 | 28 | 0.0009 | 1.41 | 99 |
| T*_min_* | 9 | 1.05 | 3.87 | 28 | 0.0006 | 1.28 | 99 |
| T*_min_* | 10 | 0.73 | 2.08 | 28 | 0.0472 | 1.66 | 95 |
| T*_min_* | 11 | 0.96 | 2.59 | 28 | 0.0149 | 1.76 | 95 |
| T*_min_* | 12 | 1.09 | 3.28 | 28 | 0.0028 | 1.58 | 99 |
| T*_max_* | 1 | 0.90 | 1.89 | 28 | 0.0686 | 2.26 | 90 |
| T*_max_* | 2 | 1.15 | 2.24 | 28 | 0.0330 | 2.43 | 95 |
| T*_max_* | 3 | 0.88 | 2.99 | 28 | 0.0057 | 1.40 | 99 |
| T*_max_* | 4 | 0.68 | 1.99 | 28 | 0.0568 | 1.63 | 90 |
| T*_max_* | 5 | 0.41 | 1.68 | 28 | 0.1048 | 1.16 | 85 |
| T*_max_* | 6 | 0.24 | 1.35 | 28 | 0.1870 | 0.85 | 80 |
| T*_max_* | 7 | 0.72 | 2.95 | 28 | 0.0064 | 1.16 | 99 |
| T*_max_* | 8 | 0.76 | 3.00 | 28 | 0.0057 | 1.20 | 99 |
| T*_max_* | 9 | 0.21 | 0.93 | 28 | 0.3627 | 1.07 | 60 |
| T*_max_* | 10 | 0.70 | 3.26 | 28 | 0.0029 | 1.01 | 99 |
| T*_max_* | 11 | 0.17 | 0.57 | 28 | 0.5722 | 1.42 | 40 |
| T*_max_* | 12 | 0.37 | 1.20 | 28 | 0.2407 | 1.47 | 75 |
| T*_mean_* | 1 | 0.91 | 2.30 | 28 | 0.0293 | 1.88 | 95 |
| T*_mean_* | 2 | 0.90 | 2.22 | 28 | 0.0349 | 1.93 | 95 |
| T*_mean_* | 3 | 0.60 | 2.91 | 28 | 0.0070 | 0.97 | 99 |
| T*_mean_* | 4 | 0.51 | 2.53 | 28 | 0.0174 | 0.95 | 95 |
| T*_mean_* | 5 | 0.36 | 2.22 | 28 | 0.0346 | 0.77 | 95 |
| T*_mean_* | 6 | 0.39 | 2.64 | 28 | 0.0134 | 0.70 | 95 |
| T*_mean_* | 7 | 0.55 | 3.07 | 28 | 0.0047 | 0.86 | 99 |
| T*_mean_* | 8 | 0.79 | 4.08 | 28 | 0.0003 | 0.91 | 99 |
| T*_mean_* | 9 | 0.59 | 3.03 | 28 | 0.0052 | 0.93 | 99 |
| T*_mean_* | 10 | 0.68 | 3.32 | 28 | 0.0025 | 0.97 | 99 |
| T*_mean_* | 11 | 0.60 | 2.24 | 28 | 0.0332 | 1.26 | 95 |
| T*_mean_* | 12 | 0.69 | 2.59 | 28 | 0.0152 | 1.26 | 95 |

**Supplementary Table 3** Test statistics for the linear trends of climate factors in March to May, August to October and full year from 1989 to 2018 in Henan Station

| Climate factor | Month | Liner trend  (d/a) | t-statistics | df | P-value | Standard error | Confidence interval  (%) |
| --- | --- | --- | --- | --- | --- | --- | --- |
| Pre | 3-5 | 0.51 | 0.96 | 27 | 0.4531 | 26.47 | 50 |
| Pre | 8-10 | 3.90 | 3.06 | 27 | 0.0091 | 59.88 | 99 |
| Pre | year | 4.00 | 2.02 | 28 | 0.0635 | 90.20 | 90 |
| Eva | 3-5 | 0.00 | -0.04 | 27 | 1.0000 | 36.74 | 0 |
| Eva | 8-10 | -2.54 | -2.51 | 27 | 0.0356 | 40.91 | 95 |
| Eva | year | -5.04 | -1.69 | 27 | 0.2227 | 127.45 | 75 |
| Eva/Pre | 3-5 | -0.02 | -0.45 | 27 | 0.6662 | 1.09 | 30 |
| Eva/Pre | 8-10 | -0.04 | -3.51 | 27 | 0.0017 | 0.47 | 99 |
| Eva/Pre | year | -0.02 | -1.65 | 27 | 0.0409 | 0.53 | 95 |
| Ssd | 3-5 | 2.86 | 2.78 | 28 | 0.0145 | 56.46 | 95 |
| Ssd | 8-10 | 0.94 | -0.02 | 27 | 0.5483 | 58.27 | 45 |
| Ssd | year | 6.61 | 1.44 | 28 | 0.1640 | 169.58 | 80 |
| H*_mean_* | 3-5 | -0.28 | -3.37 | 28 | 0.0024 | 3.33 | 99 |
| H*_mean_* | 8-10 | -0.03 | -0.81 | 28 | 0.4978 | 2.04 | 50 |
| H*_mean_* | year | -0.21 | -4.02 | 28 | 0.0012 | 2.25 | 99 |
| W*_max_* | 3-5 | -0.08 | -7.73 | 28 | 0.0000 | 0.48 | 99 |
| W*_max_* | 8-10 | -0.05 | -8.38 | 28 | 0.0000 | 0.27 | 99 |
| W*_max_* | year | -0.06 | -10.50 | 28 | 0.0000 | 0.26 | 99 |
| T*_gmax_*-T*_gmin_* | 3-5 | -0.01 | -0.37 | 28 | 0.8865 | 4.09 | 10 |
| T*_gmax_*-T*_gmin_* | 8-10 | -0.19 | -3.18 | 28 | 0.0054 | 2.96 | 99 |
| T*_gmax_*-T*_gmin_* | year | -0.21 | -3.23 | 28 | 0.0204 | 2.88 | 95 |
| T*_max_*-T*_min_* | 3-5 | 0.05 | 1.46 | 28 | 0.1249 | 1.19 | 85 |
| T*_max_*-T*_min_* | 8-10 | -0.04 | -1.65 | 28 | 0.0868 | 1.17 | 90 |
| T*_max_*-T*_min_* | year | -0.01 | -0.65 | 28 | 0.3724 | 0.93 | 60 |

**Supplementary Table 4** Basic statistics and linear trends of herbs green-up dates in Henan Station

| Herb species | Earliest green-up date  (DOY) | Latest green-up date  (DOY) | Average green-up date  (DOY) | Standard deviation (Days) | Liner trend  (d/a) | df | P value | Standard error  (days/year) | Confidence interval |
| --- | --- | --- | --- | --- | --- | --- | --- | --- | --- |
| *E. nutans* | 113 | 131 | 121 | 3.88 | 0.00 | 26 | 0.58 | 3.98 | 40% |
| *K. pygmaea* | 111 | 131 | 122 | 4.34 | 0.06 | 26 | 0.45 | 4.54 | 55% |
| *P. asiatica* | 102 | 116 | 110 | 3.57 | -0.19 | 28 | 0.18 | 3.53 | 80% |
| *P. tenuiflora* | 113 | 132 | 121 | 3.94 | -0.05 | 26 | 0.61 | 4.14 | 35% |
| *S. distigmaticus* | 111 | 130 | 123 | 4.22 | 0.08 | 26 | 0.28 | 4.26 | 70% |

**Supplementary Table 5** Basic statistics and linear trends of herbs brown-off dates in Henan Station

| Herb species | Earliest brown-off date  (DOY) | Latest brown-off date  (DOY) | Average brown-off date  (DOY) | Standard deviation (Days) | Liner trend  (d/a) | df | P value | Standard error  (days/year) | Confidence interval |
| --- | --- | --- | --- | --- | --- | --- | --- | --- | --- |
| *E. nutans* | 231 | 261 | 243 | 5.76 | -0.20 | 26 | 0.07 | 5.48 | 90% |
| *K. pygmaea* | 237 | 259 | 249 | 5.82 | 0.29 | 26 | 0.02 | 5.53 | 95% |
| *P. asiatica* | 248 | 294 | 273 | 12.29 | 1.58 | 28 | 0.00 | 6.50 | 99% |
| *P. tenuiflora* | 232 | 271 | 252 | 12.21 | 1.23 | 26 | 0.00 | 7.82 | 99% |
| *S. distigmaticus* | 227 | 282 | 260 | 16.72 | 1.84 | 26 | 0.00 | 7.22 | 99% |

**Supplementary Table 6** Basic statistics and linear trends of herbs growing season lengths in Henan Station

| Herb species | Earliest growing season lengths  (DOY) | Latest growing season lengths  (DOY) | Average growing season lengths  (DOY) | Standard deviation (Days) | Liner trend  (d/a) | df | P value | Standard error  (days/year) | Confidence interval |
| --- | --- | --- | --- | --- | --- | --- | --- | --- | --- |
| *E. nutans* | 107 | 138 | 122 | 6.32 | -0.11 | 26 | 0.20 | 6.30 | 80% |
| *K. pygmaea* | 116 | 140 | 127 | 5.54 | 0.19 | 26 | 0.26 | 5.44 | 70% |
| *P. asiatica* | 133 | 187 | 163 | 13.47 | 1.75 | 28 | 0.00 | 6.90 | 99% |
| *P. tenuiflora* | 106 | 155 | 131 | 13.14 | 1.25 | 26 | 0.00 | 8.81 | 99% |
| *S. distigmaticus* | 109 | 161 | 137 | 15.48 | 1.67 | 26 | 0.00 | 6.83 | 99% |

**Supplementary Table 7** Test statistics for the *Pearson* correlation coefficients (*r*) between 5 herbs green-up/brown-off dates and climate factors mean/accumulation values in annual mean green-up/brown-off date month and its pre three months.

| Herb Species | Phenological date | Climate factor | Month | *r* | P-value | df | Confidence interval  (%) |
| --- | --- | --- | --- | --- | --- | --- | --- |
| *E. nutans* | Green-up date | T*_gmin_* | 5 | 0.0223 | 0.9102 | 26 | 5 |
| *E. nutans* | Green-up date | T*_gmin_* | 4 | -0.2796 | 0.1495 | 26 | 85 |
| *E. nutans* | Green-up date | T*_gmin_* | 3 | -0.0878 | 0.6568 | 26 | 30 |
| *E. nutans* | Green-up date | T*_gmin_* | 2 | -0.2411 | 0.2164 | 26 | 75 |
| *E. nutans* | Green-up date | T*_gmax_* | 5 | -0.0463 | 0.8152 | 26 | 15 |
| *E. nutans* | Green-up date | T*_gmax_* | 4 | -0.0886 | 0.6538 | 26 | 30 |
| *E. nutans* | Green-up date | T*_gmax_* | 3 | -0.2770 | 0.1535 | 26 | 80 |
| *E. nutans* | Green-up date | T*_gmax_* | 2 | -0.2211 | 0.2581 | 26 | 70 |
| *E. nutans* | Green-up date | T*_gmean_* | 5 | 0.0303 | 0.8785 | 26 | 10 |
| *E. nutans* | Green-up date | T*_gmean_* | 4 | -0.1884 | 0.3371 | 26 | 65 |
| *E. nutans* | Green-up date | T*_gmean_* | 3 | -0.2505 | 0.1985 | 26 | 80 |
| *E. nutans* | Green-up date | T*_gmean_* | 2 | -0.4322 | 0.0216 | 26 | 95 |
| *E. nutans* | Green-up date | T*_min_* | 5 | 0.2118 | 0.2792 | 26 | 70 |
| *E. nutans* | Green-up date | T*_min_* | 4 | -0.1730 | 0.3786 | 26 | 60 |
| *E. nutans* | Green-up date | T*_min_* | 3 | -0.0424 | 0.8303 | 26 | 15 |
| *E. nutans* | Green-up date | T*_min_* | 2 | -0.2342 | 0.2303 | 26 | 75 |
| *E. nutans* | Green-up date | T*_max_* | 5 | 0.2137 | 0.2749 | 26 | 70 |
| *E. nutans* | Green-up date | T*_max_* | 4 | -0.1064 | 0.5900 | 26 | 40 |
| *E. nutans* | Green-up date | T*_max_* | 3 | -0.1798 | 0.3599 | 26 | 60 |
| *E. nutans* | Green-up date | T*_max_* | 2 | -0.2485 | 0.2022 | 26 | 75 |
| *E. nutans* | Green-up date | T*_mean_* | 5 | 0.2575 | 0.1859 | 26 | 80 |
| *E. nutans* | Green-up date | T*_mean_* | 4 | -0.1426 | 0.4692 | 26 | 50 |
| *E. nutans* | Green-up date | T*_mean_* | 3 | -0.1479 | 0.4526 | 26 | 50 |
| *E. nutans* | Green-up date | T*_mean_* | 2 | -0.2605 | 0.1806 | 26 | 80 |
| *E. nutans* | Green-up date | Pre | 5 | 0.1673 | 0.3948 | 26 | 60 |
| *E. nutans* | Green-up date | Pre | 4 | -0.0441 | 0.8238 | 26 | 15 |
| *E. nutans* | Green-up date | Pre | 3 | 0.0975 | 0.6218 | 26 | 35 |
| *E. nutans* | Green-up date | Pre | 2 | 0.1518 | 0.4407 | 26 | 55 |
| *E. nutans* | Green-up date | Ssd | 5 | -0.3191 | 0.0979 | 26 | 90 |
| *E. nutans* | Green-up date | Ssd | 4 | -0.0707 | 0.7206 | 26 | 25 |
| *E. nutans* | Green-up date | Ssd | 3 | -0.2266 | 0.2462 | 26 | 75 |
| *E. nutans* | Green-up date | Ssd | 2 | 0.0890 | 0.6523 | 26 | 30 |
| *E. nutans* | Green-up date | H*_min_* | 5 | 0.0937 | 0.6352 | 26 | 35 |
| *E. nutans* | Green-up date | H*_min_* | 4 | 0.1464 | 0.4573 | 26 | 50 |
| *E. nutans* | Green-up date | H*_min_* | 3 | 0.2174 | 0.2665 | 26 | 70 |
| *E. nutans* | Green-up date | H*_min_* | 2 | 0.3190 | 0.0980 | 26 | 90 |
| *E. nutans* | Green-up date | H*_mean_* | 5 | -0.0642 | 0.7456 | 26 | 25 |
| *E. nutans* | Green-up date | H*_mean_* | 4 | -0.0320 | 0.8717 | 26 | 10 |
| *E. nutans* | Green-up date | H*_mean_* | 3 | 0.1551 | 0.4306 | 26 | 55 |
| *E. nutans* | Green-up date | H*_mean_* | 2 | 0.2983 | 0.1232 | 26 | 85 |
| *E. nutans* | Green-up date | W*_mean_* | 5 | -0.0166 | 0.9332 | 26 | 5 |
| *E. nutans* | Green-up date | W*_mean_* | 4 | 0.1309 | 0.5067 | 26 | 45 |
| *E. nutans* | Green-up date | W*_mean_* | 3 | -0.0375 | 0.8498 | 26 | 15 |
| *E. nutans* | Green-up date | W*_mean_* | 2 | -0.2025 | 0.3014 | 26 | 65 |
| *E. nutans* | Green-up date | W*_max_* | 5 | 0.0010 | 0.9961 | 26 | 0 |
| *E. nutans* | Green-up date | W*_max_* | 4 | 0.0732 | 0.7112 | 26 | 25 |
| *E. nutans* | Green-up date | W*_max_* | 3 | -0.0813 | 0.6808 | 26 | 30 |
| *E. nutans* | Green-up date | W*_max_* | 2 | -0.2574 | 0.1860 | 26 | 80 |
| *E. nutans* | Green-up date | Eva | 5 | 0.2171 | 0.2672 | 26 | 70 |
| *E. nutans* | Green-up date | Eva | 4 | -0.0236 | 0.9052 | 26 | 5 |
| *E. nutans* | Green-up date | Eva | 3 | -0.2704 | 0.1641 | 26 | 80 |
| *E. nutans* | Green-up date | Eva | 2 | -0.3637 | 0.0571 | 26 | 90 |
| *K. pygmaea* | Green-up date | T*_gmin_* | 5 | 0.1904 | 0.3318 | 26 | 65 |
| *K. pygmaea* | Green-up date | T*_gmin_* | 4 | -0.1189 | 0.5467 | 26 | 45 |
| *K. pygmaea* | Green-up date | T*_gmin_* | 3 | 0.0738 | 0.7091 | 26 | 25 |
| *K. pygmaea* | Green-up date | T*_gmin_* | 2 | -0.0480 | 0.8082 | 26 | 15 |
| *K. pygmaea* | Green-up date | T*_gmax_* | 5 | 0.0904 | 0.6472 | 26 | 35 |
| *K. pygmaea* | Green-up date | T*_gmax_* | 4 | 0.1046 | 0.5965 | 26 | 40 |
| *K. pygmaea* | Green-up date | T*_gmax_* | 3 | -0.1353 | 0.4923 | 26 | 50 |
| *K. pygmaea* | Green-up date | T*_gmax_* | 2 | -0.2215 | 0.2573 | 26 | 70 |
| *K. pygmaea* | Green-up date | T*_gmean_* | 5 | 0.1989 | 0.3103 | 26 | 65 |
| *K. pygmaea* | Green-up date | T*_gmean_* | 4 | 0.0066 | 0.9733 | 26 | 1 |
| *K. pygmaea* | Green-up date | T*_gmean_* | 3 | -0.0303 | 0.8784 | 26 | 10 |
| *K. pygmaea* | Green-up date | T*_gmean_* | 2 | -0.2604 | 0.1809 | 26 | 80 |
| *K. pygmaea* | Green-up date | T*_min_* | 5 | 0.3091 | 0.1095 | 26 | 85 |
| *K. pygmaea* | Green-up date | T*_min_* | 4 | -0.0788 | 0.6903 | 26 | 30 |
| *K. pygmaea* | Green-up date | T*_min_* | 3 | 0.0233 | 0.9063 | 26 | 5 |
| *K. pygmaea* | Green-up date | T*_min_* | 2 | -0.1569 | 0.4254 | 26 | 55 |
| *K. pygmaea* | Green-up date | T*_max_* | 5 | 0.3275 | 0.0889 | 26 | 90 |
| *K. pygmaea* | Green-up date | T*_max_* | 4 | -0.0164 | 0.9340 | 26 | 5 |
| *K. pygmaea* | Green-up date | T*_max_* | 3 | -0.0728 | 0.7127 | 26 | 25 |
| *K. pygmaea* | Green-up date | T*_max_* | 2 | -0.1782 | 0.3643 | 26 | 60 |
| *K. pygmaea* | Green-up date | T*_mean_* | 5 | 0.3687 | 0.0535 | 26 | 90 |
| *K. pygmaea* | Green-up date | T*_mean_* | 4 | -0.0270 | 0.8916 | 26 | 10 |
| *K. pygmaea* | Green-up date | T*_mean_* | 3 | -0.0180 | 0.9276 | 26 | 5 |
| *K. pygmaea* | Green-up date | T*_mean_* | 2 | -0.1875 | 0.3393 | 26 | 65 |
| *K. pygmaea* | Green-up date | Pre | 5 | 0.2518 | 0.1962 | 26 | 80 |
| *K. pygmaea* | Green-up date | Pre | 4 | -0.1304 | 0.5085 | 26 | 45 |
| *K. pygmaea* | Green-up date | Pre | 3 | 0.1053 | 0.5938 | 26 | 40 |
| *K. pygmaea* | Green-up date | Pre | 2 | 0.0658 | 0.7392 | 26 | 25 |
| *K. pygmaea* | Green-up date | Ssd | 5 | -0.2446 | 0.2096 | 26 | 75 |
| *K. pygmaea* | Green-up date | Ssd | 4 | 0.1954 | 0.3189 | 26 | 65 |
| *K. pygmaea* | Green-up date | Ssd | 3 | -0.0330 | 0.8675 | 26 | 10 |
| *K. pygmaea* | Green-up date | Ssd | 2 | 0.2414 | 0.2160 | 26 | 75 |
| *K. pygmaea* | Green-up date | H*_min_* | 5 | -0.0680 | 0.7312 | 26 | 25 |
| *K. pygmaea* | Green-up date | H*_min_* | 4 | -0.0667 | 0.7358 | 26 | 25 |
| *K. pygmaea* | Green-up date | H*_min_* | 3 | 0.0391 | 0.8436 | 26 | 15 |
| *K. pygmaea* | Green-up date | H*_min_* | 2 | 0.1993 | 0.3093 | 26 | 65 |
| *K. pygmaea* | Green-up date | H*_mean_* | 5 | -0.1713 | 0.3835 | 26 | 60 |
| *K. pygmaea* | Green-up date | H*_mean_* | 4 | -0.2308 | 0.2374 | 26 | 75 |
| *K. pygmaea* | Green-up date | H*_mean_* | 3 | 0.0288 | 0.8844 | 26 | 10 |
| *K. pygmaea* | Green-up date | H*_mean_* | 2 | 0.1615 | 0.4117 | 26 | 55 |
| *K. pygmaea* | Green-up date | W*_mean_* | 5 | -0.1110 | 0.5740 | 26 | 40 |
| *K. pygmaea* | Green-up date | W*_mean_* | 4 | 0.0569 | 0.7736 | 26 | 20 |
| *K. pygmaea* | Green-up date | W*_mean_* | 3 | -0.0891 | 0.6520 | 26 | 30 |
| *K. pygmaea* | Green-up date | W*_mean_* | 2 | -0.1548 | 0.4315 | 26 | 55 |
| *K. pygmaea* | Green-up date | W*_max_* | 5 | -0.2032 | 0.2997 | 26 | 70 |
| *K. pygmaea* | Green-up date | W*_max_* | 4 | -0.0440 | 0.8242 | 26 | 15 |
| *K. pygmaea* | Green-up date | W*_max_* | 3 | -0.2848 | 0.1418 | 26 | 85 |
| *K. pygmaea* | Green-up date | W*_max_* | 2 | -0.3948 | 0.0376 | 26 | 95 |
| *K. pygmaea* | Green-up date | Eva | 5 | 0.1650 | 0.4013 | 26 | 55 |
| *K. pygmaea* | Green-up date | Eva | 4 | 0.0594 | 0.7639 | 26 | 20 |
| *K. pygmaea* | Green-up date | Eva | 3 | -0.2357 | 0.2273 | 26 | 75 |
| *K. pygmaea* | Green-up date | Eva | 2 | -0.3274 | 0.0890 | 26 | 90 |
| *P. asiatica* | Green-up date | T*_gmin_* | 4 | -0.5137 | 0.0145 | 28 | 95 |
| *P. asiatica* | Green-up date | T*_gmin_* | 3 | -0.3937 | 0.0698 | 28 | 90 |
| *P. asiatica* | Green-up date | T*_gmin_* | 2 | -0.1685 | 0.4535 | 28 | 50 |
| *P. asiatica* | Green-up date | T*_gmin_* | 1 | -0.3054 | 0.1669 | 28 | 80 |
| *P. asiatica* | Green-up date | T*_gmax_* | 4 | 0.1832 | 0.4145 | 28 | 55 |
| *P. asiatica* | Green-up date | T*_gmax_* | 3 | 0.1290 | 0.5672 | 28 | 40 |
| *P. asiatica* | Green-up date | T*_gmax_* | 2 | -0.0380 | 0.8667 | 28 | 10 |
| *P. asiatica* | Green-up date | T*_gmax_* | 1 | 0.0809 | 0.7204 | 28 | 25 |
| *P. asiatica* | Green-up date | T*_gmean_* | 4 | -0.2696 | 0.2249 | 28 | 75 |
| *P. asiatica* | Green-up date | T*_gmean_* | 3 | -0.4101 | 0.0580 | 28 | 90 |
| *P. asiatica* | Green-up date | T*_gmean_* | 2 | -0.3715 | 0.0887 | 28 | 90 |
| *P. asiatica* | Green-up date | T*_gmean_* | 1 | -0.4378 | 0.0416 | 28 | 95 |
| *P. asiatica* | Green-up date | T*_min_* | 4 | -0.5601 | 0.0067 | 28 | 99 |
| *P. asiatica* | Green-up date | T*_min_* | 3 | -0.3528 | 0.1073 | 28 | 85 |
| *P. asiatica* | Green-up date | T*_min_* | 2 | -0.0698 | 0.7576 | 28 | 20 |
| *P. asiatica* | Green-up date | T*_min_* | 1 | -0.1576 | 0.4835 | 28 | 50 |
| *P. asiatica* | Green-up date | T*_max_* | 4 | -0.2992 | 0.1762 | 28 | 80 |
| *P. asiatica* | Green-up date | T*_max_* | 3 | -0.3759 | 0.0847 | 28 | 90 |
| *P. asiatica* | Green-up date | T*_max_* | 2 | -0.2279 | 0.3076 | 28 | 65 |
| *P. asiatica* | Green-up date | T*_max_* | 1 | -0.2495 | 0.2628 | 28 | 70 |
| *P. asiatica* | Green-up date | T*_mean_* | 4 | -0.4517 | 0.0348 | 28 | 95 |
| *P. asiatica* | Green-up date | T*_mean_* | 3 | -0.4906 | 0.0204 | 28 | 95 |
| *P. asiatica* | Green-up date | T*_mean_* | 2 | -0.1852 | 0.4094 | 28 | 55 |
| *P. asiatica* | Green-up date | T*_mean_* | 1 | -0.2019 | 0.3676 | 28 | 60 |
| *P. asiatica* | Green-up date | Pre | 4 | -0.3841 | 0.0776 | 28 | 90 |
| *P. asiatica* | Green-up date | Pre | 3 | -0.1193 | 0.5969 | 28 | 40 |
| *P. asiatica* | Green-up date | Pre | 2 | 0.0927 | 0.6814 | 28 | 30 |
| *P. asiatica* | Green-up date | Pre | 1 | 0.0263 | 0.9075 | 28 | 5 |
| *P. asiatica* | Green-up date | Ssd | 4 | 0.1586 | 0.4808 | 28 | 50 |
| *P. asiatica* | Green-up date | Ssd | 3 | 0.2284 | 0.3065 | 28 | 65 |
| *P. asiatica* | Green-up date | Ssd | 2 | 0.0122 | 0.9569 | 28 | 1 |
| *P. asiatica* | Green-up date | Ssd | 1 | 0.0800 | 0.7302 | 28 | 25 |
| *P. asiatica* | Green-up date | H*_min_* | 4 | -0.0734 | 0.7456 | 28 | 25 |
| *P. asiatica* | Green-up date | H*_min_* | 3 | 0.0411 | 0.8559 | 28 | 10 |
| *P. asiatica* | Green-up date | H*_min_* | 2 | 0.3502 | 0.1101 | 28 | 85 |
| *P. asiatica* | Green-up date | H*_min_* | 1 | 0.1563 | 0.4874 | 28 | 50 |
| *P. asiatica* | Green-up date | H*_mean_* | 4 | -0.1998 | 0.3727 | 28 | 60 |
| *P. asiatica* | Green-up date | H*_mean_* | 3 | -0.0503 | 0.8241 | 28 | 15 |
| *P. asiatica* | Green-up date | H*_mean_* | 2 | 0.3820 | 0.0793 | 28 | 90 |
| *P. asiatica* | Green-up date | H*_mean_* | 1 | 0.0763 | 0.7358 | 28 | 25 |
| *P. asiatica* | Green-up date | W*_mean_* | 4 | 0.4124 | 0.0565 | 28 | 90 |
| *P. asiatica* | Green-up date | W*_mean_* | 3 | 0.2031 | 0.3647 | 28 | 60 |
| *P. asiatica* | Green-up date | W*_mean_* | 2 | -0.3737 | 0.0866 | 28 | 90 |
| *P. asiatica* | Green-up date | W*_mean_* | 1 | -0.1551 | 0.4906 | 28 | 50 |
| *P. asiatica* | Green-up date | W*_max_* | 4 | 0.4102 | 0.0579 | 28 | 90 |
| *P. asiatica* | Green-up date | W*_max_* | 3 | 0.2792 | 0.2083 | 28 | 75 |
| *P. asiatica* | Green-up date | W*_max_* | 2 | -0.2323 | 0.2982 | 28 | 70 |
| *P. asiatica* | Green-up date | W*_max_* | 1 | -0.0884 | 0.6956 | 28 | 30 |
| *P. asiatica* | Green-up date | Eva | 4 | 0.1200 | 0.5949 | 28 | 40 |
| *P. asiatica* | Green-up date | Eva | 3 | -0.0556 | 0.8057 | 28 | 15 |
| *P. asiatica* | Green-up date | Eva | 2 | -0.3824 | 0.0790 | 28 | 90 |
| *P. asiatica* | Green-up date | Eva | 1 | -0.0857 | 0.7045 | 28 | 25 |
| *P. tenuiflora* | Green-up date | T*_gmin_* | 5 | -0.0227 | 0.9088 | 26 | 5 |
| *P. tenuiflora* | Green-up date | T*_gmin_* | 4 | -0.2816 | 0.1465 | 26 | 85 |
| *P. tenuiflora* | Green-up date | T*_gmin_* | 3 | -0.1046 | 0.5964 | 26 | 40 |
| *P. tenuiflora* | Green-up date | T*_gmin_* | 2 | -0.2661 | 0.1711 | 26 | 80 |
| *P. tenuiflora* | Green-up date | T*_gmax_* | 5 | -0.0172 | 0.9308 | 26 | 5 |
| *P. tenuiflora* | Green-up date | T*_gmax_* | 4 | -0.0918 | 0.6422 | 26 | 35 |
| *P. tenuiflora* | Green-up date | T*_gmax_* | 3 | -0.2680 | 0.1679 | 26 | 80 |
| *P. tenuiflora* | Green-up date | T*_gmax_* | 2 | -0.1911 | 0.3301 | 26 | 65 |
| *P. tenuiflora* | Green-up date | T*_gmean_* | 5 | 0.0278 | 0.8882 | 26 | 10 |
| *P. tenuiflora* | Green-up date | T*_gmean_* | 4 | -0.1925 | 0.3263 | 26 | 65 |
| *P. tenuiflora* | Green-up date | T*_gmean_* | 3 | -0.2667 | 0.1701 | 26 | 80 |
| *P. tenuiflora* | Green-up date | T*_gmean_* | 2 | -0.4364 | 0.0202 | 26 | 95 |
| *P. tenuiflora* | Green-up date | T*_min_* | 5 | 0.1661 | 0.3982 | 26 | 60 |
| *P. tenuiflora* | Green-up date | T*_min_* | 4 | -0.1684 | 0.3915 | 26 | 60 |
| *P. tenuiflora* | Green-up date | T*_min_* | 3 | -0.0449 | 0.8206 | 26 | 15 |
| *P. tenuiflora* | Green-up date | T*_min_* | 2 | -0.2581 | 0.1849 | 26 | 80 |
| *P. tenuiflora* | Green-up date | T*_max_* | 5 | 0.1863 | 0.3426 | 26 | 65 |
| *P. tenuiflora* | Green-up date | T*_max_* | 4 | -0.0766 | 0.6983 | 26 | 30 |
| *P. tenuiflora* | Green-up date | T*_max_* | 3 | -0.1541 | 0.4338 | 26 | 55 |
| *P. tenuiflora* | Green-up date | T*_max_* | 2 | -0.2341 | 0.2306 | 26 | 75 |
| *P. tenuiflora* | Green-up date | T*_mean_* | 5 | 0.2237 | 0.2524 | 26 | 70 |
| *P. tenuiflora* | Green-up date | T*_mean_* | 4 | -0.1256 | 0.5244 | 26 | 45 |
| *P. tenuiflora* | Green-up date | T*_mean_* | 3 | -0.1406 | 0.4755 | 26 | 50 |
| *P. tenuiflora* | Green-up date | T*_mean_* | 2 | -0.2653 | 0.1725 | 26 | 80 |
| *P. tenuiflora* | Green-up date | Pre | 5 | 0.1170 | 0.5532 | 26 | 40 |
| *P. tenuiflora* | Green-up date | Pre | 4 | -0.0307 | 0.8767 | 26 | 10 |
| *P. tenuiflora* | Green-up date | Pre | 3 | 0.0683 | 0.7299 | 26 | 25 |
| *P. tenuiflora* | Green-up date | Pre | 2 | 0.1075 | 0.5860 | 26 | 40 |
| *P. tenuiflora* | Green-up date | Ssd | 5 | -0.3032 | 0.1168 | 26 | 85 |
| *P. tenuiflora* | Green-up date | Ssd | 4 | -0.0870 | 0.6598 | 26 | 30 |
| *P. tenuiflora* | Green-up date | Ssd | 3 | -0.2182 | 0.2646 | 26 | 70 |
| *P. tenuiflora* | Green-up date | Ssd | 2 | 0.1055 | 0.5931 | 26 | 40 |
| *P. tenuiflora* | Green-up date | H*_min_* | 5 | 0.0978 | 0.6204 | 26 | 35 |
| *P. tenuiflora* | Green-up date | H*_min_* | 4 | 0.1463 | 0.4577 | 26 | 50 |
| *P. tenuiflora* | Green-up date | H*_min_* | 3 | 0.1962 | 0.3171 | 26 | 65 |
| *P. tenuiflora* | Green-up date | H*_min_* | 2 | 0.2931 | 0.1301 | 26 | 85 |
| *P. tenuiflora* | Green-up date | H*_mean_* | 5 | -0.0798 | 0.6863 | 26 | 30 |
| *P. tenuiflora* | Green-up date | H*_mean_* | 4 | -0.0554 | 0.7797 | 26 | 20 |
| *P. tenuiflora* | Green-up date | H*_mean_* | 3 | 0.1176 | 0.5510 | 26 | 40 |
| *P. tenuiflora* | Green-up date | H*_mean_* | 2 | 0.2580 | 0.1851 | 26 | 80 |
| *P. tenuiflora* | Green-up date | W*_mean_* | 5 | -0.0286 | 0.8853 | 26 | 10 |
| *P. tenuiflora* | Green-up date | W*_mean_* | 4 | 0.1387 | 0.4815 | 26 | 50 |
| *P. tenuiflora* | Green-up date | W*_mean_* | 3 | -0.0336 | 0.8651 | 26 | 10 |
| *P. tenuiflora* | Green-up date | W*_mean_* | 2 | -0.2237 | 0.2525 | 26 | 70 |
| *P. tenuiflora* | Green-up date | W*_max_* | 5 | -0.0132 | 0.9469 | 26 | 5 |
| *P. tenuiflora* | Green-up date | W*_max_* | 4 | 0.1099 | 0.5777 | 26 | 40 |
| *P. tenuiflora* | Green-up date | W*_max_* | 3 | -0.0288 | 0.8844 | 26 | 10 |
| *P. tenuiflora* | Green-up date | W*_max_* | 2 | -0.2096 | 0.2845 | 26 | 70 |
| *P. tenuiflora* | Green-up date | Eva | 5 | 0.2484 | 0.2025 | 26 | 75 |
| *P. tenuiflora* | Green-up date | Eva | 4 | 0.0269 | 0.8918 | 26 | 10 |
| *P. tenuiflora* | Green-up date | Eva | 3 | -0.2129 | 0.2766 | 26 | 70 |
| *P. tenuiflora* | Green-up date | Eva | 2 | -0.3212 | 0.0955 | 26 | 90 |
| *S. distigmaticus* | Green-up date | T*_gmin_* | 5 | 0.2757 | 0.1556 | 26 | 80 |
| *S. distigmaticus* | Green-up date | T*_gmin_* | 4 | -0.0032 | 0.9873 | 26 | 1 |
| *S. distigmaticus* | Green-up date | T*_gmin_* | 3 | 0.0955 | 0.6290 | 26 | 35 |
| *S. distigmaticus* | Green-up date | T*_gmin_* | 2 | -0.0235 | 0.9055 | 26 | 5 |
| *S. distigmaticus* | Green-up date | T*_gmax_* | 5 | 0.1098 | 0.5782 | 26 | 40 |
| *S. distigmaticus* | Green-up date | T*_gmax_* | 4 | 0.1641 | 0.4040 | 26 | 55 |
| *S. distigmaticus* | Green-up date | T*_gmax_* | 3 | -0.0354 | 0.8580 | 26 | 10 |
| *S. distigmaticus* | Green-up date | T*_gmax_* | 2 | -0.1010 | 0.6089 | 26 | 35 |
| *S. distigmaticus* | Green-up date | T*_gmean_* | 5 | 0.2560 | 0.1885 | 26 | 80 |
| *S. distigmaticus* | Green-up date | T*_gmean_* | 4 | 0.0941 | 0.6337 | 26 | 35 |
| *S. distigmaticus* | Green-up date | T*_gmean_* | 3 | 0.0488 | 0.8051 | 26 | 15 |
| *S. distigmaticus* | Green-up date | T*_gmean_* | 2 | -0.1471 | 0.4552 | 26 | 50 |
| *S. distigmaticus* | Green-up date | T*_min_* | 5 | 0.3456 | 0.0717 | 26 | 90 |
| *S. distigmaticus* | Green-up date | T*_min_* | 4 | 0.0259 | 0.8961 | 26 | 10 |
| *S. distigmaticus* | Green-up date | T*_min_* | 3 | -0.0051 | 0.9796 | 26 | 1 |
| *S. distigmaticus* | Green-up date | T*_min_* | 2 | -0.0936 | 0.6356 | 26 | 35 |
| *S. distigmaticus* | Green-up date | T*_max_* | 5 | 0.3585 | 0.0610 | 26 | 90 |
| *S. distigmaticus* | Green-up date | T*_max_* | 4 | 0.0721 | 0.7154 | 26 | 25 |
| *S. distigmaticus* | Green-up date | T*_max_* | 3 | 0.0040 | 0.9838 | 26 | 1 |
| *S. distigmaticus* | Green-up date | T*_max_* | 2 | -0.0658 | 0.7392 | 26 | 25 |
| *S. distigmaticus* | Green-up date | T*_mean_* | 5 | 0.4062 | 0.0320 | 26 | 95 |
| *S. distigmaticus* | Green-up date | T*_mean_* | 4 | 0.0812 | 0.6813 | 26 | 30 |
| *S. distigmaticus* | Green-up date | T*_mean_* | 3 | 0.0195 | 0.9214 | 26 | 5 |
| *S. distigmaticus* | Green-up date | T*_mean_* | 2 | -0.0915 | 0.6433 | 26 | 35 |
| *S. distigmaticus* | Green-up date | Pre | 5 | 0.3101 | 0.1083 | 26 | 85 |
| *S. distigmaticus* | Green-up date | Pre | 4 | -0.0902 | 0.6479 | 26 | 35 |
| *S. distigmaticus* | Green-up date | Pre | 3 | 0.1111 | 0.5737 | 26 | 40 |
| *S. distigmaticus* | Green-up date | Pre | 2 | -0.0841 | 0.6704 | 26 | 30 |
| *S. distigmaticus* | Green-up date | Ssd | 5 | -0.1676 | 0.3939 | 26 | 60 |
| *S. distigmaticus* | Green-up date | Ssd | 4 | 0.2840 | 0.1430 | 26 | 85 |
| *S. distigmaticus* | Green-up date | Ssd | 3 | 0.0347 | 0.8607 | 26 | 10 |
| *S. distigmaticus* | Green-up date | Ssd | 2 | 0.2757 | 0.1556 | 26 | 80 |
| *S. distigmaticus* | Green-up date | H*_min_* | 5 | -0.1147 | 0.5613 | 26 | 40 |
| *S. distigmaticus* | Green-up date | H*_min_* | 4 | -0.0856 | 0.6649 | 26 | 30 |
| *S. distigmaticus* | Green-up date | H*_min_* | 3 | -0.0543 | 0.7838 | 26 | 20 |
| *S. distigmaticus* | Green-up date | H*_min_* | 2 | 0.0568 | 0.7741 | 26 | 20 |
| *S. distigmaticus* | Green-up date | H*_mean_* | 5 | -0.2117 | 0.2796 | 26 | 70 |
| *S. distigmaticus* | Green-up date | H*_mean_* | 4 | -0.2822 | 0.1457 | 26 | 85 |
| *S. distigmaticus* | Green-up date | H*_mean_* | 3 | -0.0784 | 0.6917 | 26 | 30 |
| *S. distigmaticus* | Green-up date | H*_mean_* | 2 | 0.0107 | 0.9568 | 26 | 1 |
| *S. distigmaticus* | Green-up date | W*_mean_* | 5 | -0.1416 | 0.4721 | 26 | 50 |
| *S. distigmaticus* | Green-up date | W*_mean_* | 4 | -0.0151 | 0.9390 | 26 | 5 |
| *S. distigmaticus* | Green-up date | W*_mean_* | 3 | -0.0651 | 0.7420 | 26 | 25 |
| *S. distigmaticus* | Green-up date | W*_mean_* | 2 | -0.1725 | 0.3799 | 26 | 60 |
| *S. distigmaticus* | Green-up date | W*_max_* | 5 | -0.3147 | 0.1029 | 26 | 85 |
| *S. distigmaticus* | Green-up date | W*_max_* | 4 | -0.0697 | 0.7246 | 26 | 25 |
| *S. distigmaticus* | Green-up date | W*_max_* | 3 | -0.2767 | 0.1540 | 26 | 80 |
| *S. distigmaticus* | Green-up date | W*_max_* | 2 | -0.3758 | 0.0488 | 26 | 95 |
| *S. distigmaticus* | Green-up date | Eva | 5 | 0.1774 | 0.3665 | 26 | 60 |
| *S. distigmaticus* | Green-up date | Eva | 4 | 0.1397 | 0.4785 | 26 | 50 |
| *S. distigmaticus* | Green-up date | Eva | 3 | -0.1381 | 0.4835 | 26 | 50 |
| *S. distigmaticus* | Green-up date | Eva | 2 | -0.2283 | 0.2426 | 26 | 75 |
| *E. nutans* | Brown-off date | T*_gmin_* | 8 | -0.1133 | 0.5661 | 26 | 40 |
| *E. nutans* | Brown-off date | T*_gmin_* | 7 | -0.2657 | 0.1717 | 26 | 80 |
| *E. nutans* | Brown-off date | T*_gmin_* | 6 | -0.2671 | 0.1694 | 26 | 80 |
| *E. nutans* | Brown-off date | T*_gmin_* | 5 | -0.1810 | 0.3565 | 26 | 60 |
| *E. nutans* | Brown-off date | T*_gmax_* | 8 | -0.1538 | 0.4346 | 26 | 55 |
| *E. nutans* | Brown-off date | T*_gmax_* | 7 | -0.2193 | 0.2623 | 26 | 70 |
| *E. nutans* | Brown-off date | T*_gmax_* | 6 | -0.4462 | 0.0173 | 26 | 95 |
| *E. nutans* | Brown-off date | T*_gmax_* | 5 | -0.0281 | 0.8873 | 26 | 10 |
| *E. nutans* | Brown-off date | T*_gmean_* | 8 | -0.2710 | 0.1630 | 26 | 80 |
| *E. nutans* | Brown-off date | T*_gmean_* | 7 | -0.3413 | 0.0755 | 26 | 90 |
| *E. nutans* | Brown-off date | T*_gmean_* | 6 | -0.5578 | 0.0020 | 26 | 99 |
| *E. nutans* | Brown-off date | T*_gmean_* | 5 | -0.1941 | 0.3224 | 26 | 65 |
| *E. nutans* | Brown-off date | T*_min_* | 8 | 0.0068 | 0.9726 | 26 | 1 |
| *E. nutans* | Brown-off date | T*_min_* | 7 | -0.1423 | 0.4701 | 26 | 50 |
| *E. nutans* | Brown-off date | T*_min_* | 6 | -0.2629 | 0.1766 | 26 | 80 |
| *E. nutans* | Brown-off date | T*_min_* | 5 | -0.0129 | 0.9481 | 26 | 5 |
| *E. nutans* | Brown-off date | T*_max_* | 8 | -0.3136 | 0.1042 | 26 | 85 |
| *E. nutans* | Brown-off date | T*_max_* | 7 | -0.4626 | 0.0132 | 26 | 95 |
| *E. nutans* | Brown-off date | T*_max_* | 6 | -0.5970 | 0.0008 | 26 | 99 |
| *E. nutans* | Brown-off date | T*_max_* | 5 | -0.2999 | 0.1210 | 26 | 85 |
| *E. nutans* | Brown-off date | T*_mean_* | 8 | -0.2156 | 0.2704 | 26 | 70 |
| *E. nutans* | Brown-off date | T*_mean_* | 7 | -0.3472 | 0.0702 | 26 | 90 |
| *E. nutans* | Brown-off date | T*_mean_* | 6 | -0.5152 | 0.0050 | 26 | 99 |
| *E. nutans* | Brown-off date | T*_mean_* | 5 | -0.2301 | 0.2388 | 26 | 75 |
| *E. nutans* | Brown-off date | Pre | 8 | -0.3021 | 0.1182 | 26 | 85 |
| *E. nutans* | Brown-off date | Pre | 7 | 0.1430 | 0.4678 | 26 | 50 |
| *E. nutans* | Brown-off date | Pre | 6 | 0.6573 | 0.0001 | 26 | 99 |
| *E. nutans* | Brown-off date | Pre | 5 | -0.2487 | 0.2020 | 26 | 75 |
| *E. nutans* | Brown-off date | Ssd | 8 | -0.1924 | 0.3267 | 26 | 65 |
| *E. nutans* | Brown-off date | Ssd | 7 | -0.1220 | 0.5361 | 26 | 75 |
| *E. nutans* | Brown-off date | Ssd | 6 | -0.2553 | 0.1897 | 26 | 80 |
| *E. nutans* | Brown-off date | Ssd | 5 | -0.2220 | 0.2562 | 26 | 70 |
| *E. nutans* | Brown-off date | H*_min_* | 8 | 0.3587 | 0.0608 | 26 | 90 |
| *E. nutans* | Brown-off date | H*_min_* | 7 | 0.3139 | 0.1038 | 26 | 85 |
| *E. nutans* | Brown-off date | H*_min_* | 6 | 0.3317 | 0.0846 | 26 | 90 |
| *E. nutans* | Brown-off date | H*_min_* | 5 | 0.1746 | 0.3743 | 26 | 60 |
| *E. nutans* | Brown-off date | H*_mean_* | 8 | 0.3511 | 0.0670 | 26 | 90 |
| *E. nutans* | Brown-off date | H*_mean_* | 7 | 0.1367 | 0.4879 | 26 | 50 |
| *E. nutans* | Brown-off date | H*_mean_* | 6 | 0.2636 | 0.1754 | 26 | 80 |
| *E. nutans* | Brown-off date | H*_mean_* | 5 | 0.1250 | 0.5263 | 26 | 45 |
| *E. nutans* | Brown-off date | W*_mean_* | 8 | 0.0958 | 0.6278 | 26 | 35 |
| *E. nutans* | Brown-off date | W*_mean_* | 7 | 0.1937 | 0.3233 | 26 | 65 |
| *E. nutans* | Brown-off date | W*_mean_* | 6 | 0.0868 | 0.6603 | 26 | 30 |
| *E. nutans* | Brown-off date | W*_mean_* | 5 | 0.3070 | 0.1121 | 26 | 85 |
| *E. nutans* | Brown-off date | W*_max_* | 8 | 0.4888 | 0.0083 | 26 | 99 |
| *E. nutans* | Brown-off date | W*_max_* | 7 | 0.2959 | 0.1264 | 26 | 85 |
| *E. nutans* | Brown-off date | W*_max_* | 6 | 0.1773 | 0.3668 | 26 | 60 |
| *E. nutans* | Brown-off date | W*_max_* | 5 | -0.0197 | 0.9208 | 26 | 5 |
| *E. nutans* | Brown-off date | Eva | 8 | -0.0911 | 0.6447 | 26 | 35 |
| *E. nutans* | Brown-off date | Eva | 7 | -0.0171 | 0.9312 | 26 | 5 |
| *E. nutans* | Brown-off date | Eva | 6 | -0.0318 | 0.8726 | 26 | 10 |
| *E. nutans* | Brown-off date | Eva | 5 | 0.0396 | 0.8414 | 26 | 15 |
| *K. pygmaea* | Brown-off date | T*_gmin_* | 9 | 0.3653 | 0.0560 | 26 | 90 |
| *K. pygmaea* | Brown-off date | T*_gmin_* | 8 | 0.3906 | 0.0399 | 26 | 95 |
| *K. pygmaea* | Brown-off date | T*_gmin_* | 7 | 0.3388 | 0.0778 | 26 | 90 |
| *K. pygmaea* | Brown-off date | T*_gmin_* | 6 | 0.3309 | 0.0854 | 26 | 90 |
| *K. pygmaea* | Brown-off date | T*_gmax_* | 9 | 0.1516 | 0.4413 | 26 | 55 |
| *K. pygmaea* | Brown-off date | T*_gmax_* | 8 | -0.1254 | 0.5247 | 26 | 45 |
| *K. pygmaea* | Brown-off date | T*_gmax_* | 7 | -0.0762 | 0.7000 | 26 | 30 |
| *K. pygmaea* | Brown-off date | T*_gmax_* | 6 | 0.0638 | 0.7472 | 26 | 25 |
| *K. pygmaea* | Brown-off date | T*_gmean_* | 9 | 0.4203 | 0.0260 | 26 | 95 |
| *K. pygmaea* | Brown-off date | T*_gmean_* | 8 | 0.0444 | 0.8226 | 26 | 15 |
| *K. pygmaea* | Brown-off date | T*_gmean_* | 7 | 0.0194 | 0.9220 | 26 | 5 |
| *K. pygmaea* | Brown-off date | T*_gmean_* | 6 | 0.1126 | 0.5685 | 26 | 40 |
| *K. pygmaea* | Brown-off date | T*_min_* | 9 | 0.2818 | 0.1463 | 26 | 85 |
| *K. pygmaea* | Brown-off date | T*_min_* | 8 | 0.3409 | 0.0759 | 26 | 90 |
| *K. pygmaea* | Brown-off date | T*_min_* | 7 | 0.2585 | 0.1841 | 26 | 80 |
| *K. pygmaea* | Brown-off date | T*_min_* | 6 | 0.1381 | 0.4833 | 26 | 50 |
| *K. pygmaea* | Brown-off date | T*_max_* | 9 | 0.3603 | 0.0596 | 26 | 90 |
| *K. pygmaea* | Brown-off date | T*_max_* | 8 | -0.0048 | 0.9808 | 26 | 1 |
| *K. pygmaea* | Brown-off date | T*_max_* | 7 | -0.0187 | 0.9247 | 26 | 5 |
| *K. pygmaea* | Brown-off date | T*_max_* | 6 | -0.1751 | 0.3730 | 26 | 5 |
| *K. pygmaea* | Brown-off date | T*_mean_* | 9 | 0.3804 | 0.0458 | 26 | 95 |
| *K. pygmaea* | Brown-off date | T*_mean_* | 8 | 0.1662 | 0.3979 | 26 | 60 |
| *K. pygmaea* | Brown-off date | T*_mean_* | 7 | 0.1582 | 0.4213 | 26 | 55 |
| *K. pygmaea* | Brown-off date | T*_mean_* | 6 | -0.0088 | 0.9645 | 26 | 3 |
| *K. pygmaea* | Brown-off date | Pre | 9 | 0.4266 | 0.0236 | 26 | 95 |
| *K. pygmaea* | Brown-off date | Pre | 8 | 0.0333 | 0.8665 | 26 | 10 |
| *K. pygmaea* | Brown-off date | Pre | 7 | 0.2590 | 0.1832 | 26 | 80 |
| *K. pygmaea* | Brown-off date | Pre | 6 | 0.3349 | 0.0815 | 26 | 90 |
| *K. pygmaea* | Brown-off date | Ssd | 9 | 0.1855 | 0.3446 | 26 | 65 |
| *K. pygmaea* | Brown-off date | Ssd | 8 | -0.1228 | 0.5337 | 26 | 45 |
| *K. pygmaea* | Brown-off date | Ssd | 7 | -0.2148 | 0.2723 | 26 | 70 |
| *K. pygmaea* | Brown-off date | Ssd | 6 | -0.1327 | 0.5009 | 26 | 45 |
| *K. pygmaea* | Brown-off date | H*_min_* | 9 | -0.0223 | 0.9103 | 26 | 5 |
| *K. pygmaea* | Brown-off date | H*_min_* | 8 | 0.1205 | 0.5412 | 26 | 45 |
| *K. pygmaea* | Brown-off date | H*_min_* | 7 | 0.0612 | 0.7570 | 26 | 20 |
| *K. pygmaea* | Brown-off date | H*_min_* | 6 | -0.1067 | 0.5890 | 26 | 40 |
| *K. pygmaea* | Brown-off date | H*_mean_* | 9 | -0.1419 | 0.4714 | 26 | 50 |
| *K. pygmaea* | Brown-off date | H*_mean_* | 8 | 0.0795 | 0.6876 | 26 | 30 |
| *K. pygmaea* | Brown-off date | H*_mean_* | 7 | -0.1003 | 0.6117 | 26 | 35 |
| *K. pygmaea* | Brown-off date | H*_mean_* | 6 | -0.1364 | 0.4890 | 26 | 50 |
| *K. pygmaea* | Brown-off date | W*_mean_* | 9 | 0.1708 | 0.3848 | 26 | 60 |
| *K. pygmaea* | Brown-off date | W*_mean_* | 8 | 0.1866 | 0.3417 | 26 | 65 |
| *K. pygmaea* | Brown-off date | W*_mean_* | 7 | 0.0823 | 0.6771 | 26 | 30 |
| *K. pygmaea* | Brown-off date | W*_mean_* | 6 | -0.1696 | 0.3883 | 26 | 60 |
| *K. pygmaea* | Brown-off date | W*_max_* | 9 | -0.0211 | 0.9153 | 26 | 5 |
| *K. pygmaea* | Brown-off date | W*_max_* | 8 | -0.1447 | 0.4624 | 26 | 50 |
| *K. pygmaea* | Brown-off date | W*_max_* | 7 | -0.2865 | 0.1393 | 26 | 85 |
| *K. pygmaea* | Brown-off date | W*_max_* | 6 | -0.4543 | 0.0152 | 26 | 95 |
| *K. pygmaea* | Brown-off date | Eva | 9 | -0.4306 | 0.0222 | 26 | 95 |
| *K. pygmaea* | Brown-off date | Eva | 8 | -0.6207 | 0.0004 | 26 | 99 |
| *K. pygmaea* | Brown-off date | Eva | 7 | -0.5188 | 0.0047 | 26 | 99 |
| *K. pygmaea* | Brown-off date | Eva | 6 | -0.5089 | 0.0057 | 26 | 99 |
| *P. asiatica* | Brown-off date | T*_gmin_* | 9 | 0.8394 | 0.0000 | 28 | 99 |
| *P. asiatica* | Brown-off date | T*_gmin_* | 8 | 0.8125 | 0.0000 | 28 | 99 |
| *P. asiatica* | Brown-off date | T*_gmin_* | 7 | 0.5596 | 0.0068 | 28 | 99 |
| *P. asiatica* | Brown-off date | T*_gmin_* | 6 | 0.5157 | 0.0140 | 28 | 95 |
| *P. asiatica* | Brown-off date | T*_gmax_* | 9 | -0.2996 | 0.1755 | 28 | 80 |
| *P. asiatica* | Brown-off date | T*_gmax_* | 8 | -0.1547 | 0.4919 | 28 | 50 |
| *P. asiatica* | Brown-off date | T*_gmax_* | 7 | -0.1607 | 0.4749 | 28 | 50 |
| *P. asiatica* | Brown-off date | T*_gmax_* | 6 | -0.1381 | 0.5399 | 28 | 45 |
| *P. asiatica* | Brown-off date | T*_gmean_* | 9 | 0.3721 | 0.0881 | 28 | 90 |
| *P. asiatica* | Brown-off date | T*_gmean_* | 8 | 0.3661 | 0.0938 | 28 | 90 |
| *P. asiatica* | Brown-off date | T*_gmean_* | 7 | 0.0986 | 0.6626 | 28 | 30 |
| *P. asiatica* | Brown-off date | T*_gmean_* | 6 | -0.0211 | 0.9257 | 28 | 5 |
| *P. asiatica* | Brown-off date | T*_min_* | 9 | 0.6811 | 0.0005 | 28 | 99 |
| *P. asiatica* | Brown-off date | T*_min_* | 8 | 0.6879 | 0.0004 | 28 | 99 |
| *P. asiatica* | Brown-off date | T*_min_* | 7 | 0.3770 | 0.0837 | 28 | 90 |
| *P. asiatica* | Brown-off date | T*_min_* | 6 | 0.2730 | 0.2190 | 28 | 75 |
| *P. asiatica* | Brown-off date | T*_max_* | 9 | 0.1486 | 0.5093 | 28 | 45 |
| *P. asiatica* | Brown-off date | T*_max_* | 8 | 0.3292 | 0.1346 | 28 | 85 |
| *P. asiatica* | Brown-off date | T*_max_* | 7 | 0.1247 | 0.5804 | 28 | 40 |
| *P. asiatica* | Brown-off date | T*_max_* | 6 | -0.1462 | 0.5161 | 28 | 45 |
| *P. asiatica* | Brown-off date | T*_mean_* | 9 | 0.5434 | 0.0090 | 28 | 99 |
| *P. asiatica* | Brown-off date | T*_mean_* | 8 | 0.5960 | 0.0034 | 28 | 99 |
| *P. asiatica* | Brown-off date | T*_mean_* | 7 | 0.3247 | 0.1404 | 28 | 85 |
| *P. asiatica* | Brown-off date | T*_mean_* | 6 | -0.0476 | 0.8335 | 28 | 15 |
| *P. asiatica* | Brown-off date | Pre | 9 | 0.2964 | 0.1805 | 28 | 80 |
| *P. asiatica* | Brown-off date | Pre | 8 | 0.5102 | 0.0153 | 28 | 95 |
| *P. asiatica* | Brown-off date | Pre | 7 | 0.2888 | 0.1923 | 28 | 80 |
| *P. asiatica* | Brown-off date | Pre | 6 | 0.2012 | 0.3691 | 28 | 60 |
| *P. asiatica* | Brown-off date | Ssd | 9 | -0.3628 | 0.0970 | 28 | 90 |
| *P. asiatica* | Brown-off date | Ssd | 8 | -0.2270 | 0.3097 | 28 | 65 |
| *P. asiatica* | Brown-off date | Ssd | 7 | -0.1513 | 0.5015 | 28 | 45 |
| *P. asiatica* | Brown-off date | Ssd | 6 | -0.0842 | 0.7096 | 28 | 25 |
| *P. asiatica* | Brown-off date | H*_min_* | 9 | 0.1003 | 0.6569 | 28 | 30 |
| *P. asiatica* | Brown-off date | H*_min_* | 8 | -0.0519 | 0.8187 | 28 | 15 |
| *P. asiatica* | Brown-off date | H*_min_* | 7 | -0.1806 | 0.4213 | 28 | 55 |
| *P. asiatica* | Brown-off date | H*_min_* | 6 | -0.3023 | 0.1715 | 28 | 80 |
| *P. asiatica* | Brown-off date | H*_mean_* | 9 | 0.1876 | 0.4032 | 28 | 55 |
| *P. asiatica* | Brown-off date | H*_mean_* | 8 | -0.0826 | 0.7148 | 28 | 25 |
| *P. asiatica* | Brown-off date | H*_mean_* | 7 | -0.2506 | 0.2606 | 28 | 70 |
| *P. asiatica* | Brown-off date | H*_mean_* | 6 | -0.2213 | 0.3224 | 28 | 65 |
| *P. asiatica* | Brown-off date | W*_mean_* | 9 | 0.4413 | 0.0398 | 28 | 95 |
| *P. asiatica* | Brown-off date | W*_mean_* | 8 | 0.7674 | 0.0000 | 28 | 100 |
| *P. asiatica* | Brown-off date | W*_mean_* | 7 | 0.7424 | 0.0001 | 28 | 99 |
| *P. asiatica* | Brown-off date | W*_mean_* | 6 | 0.4675 | 0.0282 | 28 | 95 |
| *P. asiatica* | Brown-off date | W*_max_* | 9 | -0.4518 | 0.0348 | 28 | 95 |
| *P. asiatica* | Brown-off date | W*_max_* | 8 | -0.5642 | 0.0062 | 28 | 99 |
| *P. asiatica* | Brown-off date | W*_max_* | 7 | -0.4611 | 0.0308 | 28 | 95 |
| *P. asiatica* | Brown-off date | W*_max_* | 6 | -0.5854 | 0.0042 | 28 | 99 |
| *P. asiatica* | Brown-off date | Eva | 9 | -0.6683 | 0.0007 | 28 | 99 |
| *P. asiatica* | Brown-off date | Eva | 8 | -0.3719 | 0.0883 | 28 | 90 |
| *P. asiatica* | Brown-off date | Eva | 7 | -0.3088 | 0.1620 | 28 | 80 |
| *P. asiatica* | Brown-off date | Eva | 6 | -0.4515 | 0.0349 | 28 | 95 |
| *P. tenuiflora* | Brown-off date | T*_gmin_* | 9 | 0.7109 | 0.0000 | 26 | 99 |
| *P. tenuiflora* | Brown-off date | T*_gmin_* | 8 | 0.6989 | 0.0000 | 26 | 99 |
| *P. tenuiflora* | Brown-off date | T*_gmin_* | 7 | 0.5282 | 0.0039 | 26 | 99 |
| *P. tenuiflora* | Brown-off date | T*_gmin_* | 6 | 0.6584 | 0.0001 | 26 | 99 |
| *P. tenuiflora* | Brown-off date | T*_gmax_* | 9 | -0.0930 | 0.6378 | 26 | 35 |
| *P. tenuiflora* | Brown-off date | T*_gmax_* | 8 | 0.1349 | 0.4936 | 26 | 50 |
| *P. tenuiflora* | Brown-off date | T*_gmax_* | 7 | 0.0138 | 0.9443 | 26 | 5 |
| *P. tenuiflora* | Brown-off date | T*_gmax_* | 6 | -0.0677 | 0.7321 | 26 | 25 |
| *P. tenuiflora* | Brown-off date | T*_gmean_* | 9 | 0.5031 | 0.0064 | 26 | 99 |
| *P. tenuiflora* | Brown-off date | T*_gmean_* | 8 | 0.3909 | 0.0397 | 26 | 95 |
| *P. tenuiflora* | Brown-off date | T*_gmean_* | 7 | 0.2202 | 0.2602 | 26 | 70 |
| *P. tenuiflora* | Brown-off date | T*_gmean_* | 6 | 0.2573 | 0.1862 | 26 | 80 |
| *P. tenuiflora* | Brown-off date | T*_min_* | 9 | 0.5606 | 0.0019 | 26 | 99 |
| *P. tenuiflora* | Brown-off date | T*_min_* | 8 | 0.5311 | 0.0036 | 26 | 99 |
| *P. tenuiflora* | Brown-off date | T*_min_* | 7 | 0.3349 | 0.0815 | 26 | 90 |
| *P. tenuiflora* | Brown-off date | T*_min_* | 6 | 0.4590 | 0.0140 | 26 | 95 |
| *P. tenuiflora* | Brown-off date | T*_max_* | 9 | 0.2318 | 0.2353 | 26 | 75 |
| *P. tenuiflora* | Brown-off date | T*_max_* | 8 | 0.3188 | 0.0982 | 26 | 90 |
| *P. tenuiflora* | Brown-off date | T*_max_* | 7 | 0.1185 | 0.5482 | 26 | 45 |
| *P. tenuiflora* | Brown-off date | T*_max_* | 6 | -0.0418 | 0.8328 | 26 | 15 |
| *P. tenuiflora* | Brown-off date | T*_mean_* | 9 | 0.4875 | 0.0085 | 26 | 99 |
| *P. tenuiflora* | Brown-off date | T*_mean_* | 8 | 0.4614 | 0.0135 | 26 | 95 |
| *P. tenuiflora* | Brown-off date | T*_mean_* | 7 | 0.2775 | 0.1528 | 26 | 80 |
| *P. tenuiflora* | Brown-off date | T*_mean_* | 6 | 0.2111 | 0.2808 | 26 | 70 |
| *P. tenuiflora* | Brown-off date | Pre | 9 | 0.3093 | 0.1093 | 26 | 85 |
| *P. tenuiflora* | Brown-off date | Pre | 8 | 0.1866 | 0.3418 | 26 | 65 |
| *P. tenuiflora* | Brown-off date | Pre | 7 | 0.0815 | 0.6803 | 26 | 30 |
| *P. tenuiflora* | Brown-off date | Pre | 6 | 0.2765 | 0.1544 | 26 | 80 |
| *P. tenuiflora* | Brown-off date | Ssd | 9 | -0.1105 | 0.5755 | 26 | 40 |
| *P. tenuiflora* | Brown-off date | Ssd | 8 | 0.0734 | 0.7106 | 26 | 25 |
| *P. tenuiflora* | Brown-off date | Ssd | 7 | -0.0222 | 0.9106 | 26 | 5 |
| *P. tenuiflora* | Brown-off date | Ssd | 6 | -0.1256 | 0.5241 | 26 | 45 |
| *P. tenuiflora* | Brown-off date | H*_min_* | 9 | 0.0082 | 0.9671 | 26 | 1 |
| *P. tenuiflora* | Brown-off date | H*_min_* | 8 | -0.2829 | 0.1447 | 26 | 85 |
| *P. tenuiflora* | Brown-off date | H*_min_* | 7 | -0.2396 | 0.2194 | 26 | 75 |
| *P. tenuiflora* | Brown-off date | H*_min_* | 6 | -0.2388 | 0.2210 | 26 | 75 |
| *P. tenuiflora* | Brown-off date | H*_mean_* | 9 | -0.0612 | 0.7569 | 26 | 20 |
| *P. tenuiflora* | Brown-off date | H*_mean_* | 8 | -0.2794 | 0.1499 | 26 | 85 |
| *P. tenuiflora* | Brown-off date | H*_mean_* | 7 | -0.3158 | 0.1017 | 26 | 85 |
| *P. tenuiflora* | Brown-off date | H*_mean_* | 6 | -0.2904 | 0.1338 | 26 | 85 |
| *P. tenuiflora* | Brown-off date | W*_mean_* | 9 | 0.2565 | 0.1876 | 26 | 80 |
| *P. tenuiflora* | Brown-off date | W*_mean_* | 8 | 0.1918 | 0.3283 | 26 | 65 |
| *P. tenuiflora* | Brown-off date | W*_mean_* | 7 | 0.1758 | 0.3709 | 26 | 60 |
| *P. tenuiflora* | Brown-off date | W*_mean_* | 6 | -0.0611 | 0.7573 | 26 | 20 |
| *P. tenuiflora* | Brown-off date | W*_max_* | 9 | -0.4019 | 0.0340 | 26 | 95 |
| *P. tenuiflora* | Brown-off date | W*_max_* | 8 | -0.5151 | 0.0050 | 26 | 99 |
| *P. tenuiflora* | Brown-off date | W*_max_* | 7 | -0.6018 | 0.0007 | 26 | 99 |
| *P. tenuiflora* | Brown-off date | W*_max_* | 6 | -0.6826 | 0.0001 | 26 | 99 |
| *P. tenuiflora* | Brown-off date | Eva | 9 | -0.7043 | 0.0000 | 26 | 99 |
| *P. tenuiflora* | Brown-off date | Eva | 8 | -0.5808 | 0.0012 | 26 | 99 |
| *P. tenuiflora* | Brown-off date | Eva | 7 | -0.5818 | 0.0012 | 26 | 99 |
| *P. tenuiflora* | Brown-off date | Eva | 6 | -0.6080 | 0.0006 | 26 | 99 |
| *S. distigmaticus* | Brown-off date | T*_gmin_* | 9 | 0.7530 | 0.0000 | 26 | 99 |
| *S. distigmaticus* | Brown-off date | T*_gmin_* | 8 | 0.7497 | 0.0000 | 26 | 99 |
| *S. distigmaticus* | Brown-off date | T*_gmin_* | 7 | 0.5947 | 0.0008 | 26 | 99 |
| *S. distigmaticus* | Brown-off date | T*_gmin_* | 6 | 0.7329 | 0.0000 | 26 | 99 |
| *S. distigmaticus* | Brown-off date | T*_gmax_* | 9 | -0.0448 | 0.8211 | 26 | 15 |
| *S. distigmaticus* | Brown-off date | T*_gmax_* | 8 | 0.2076 | 0.2892 | 26 | 70 |
| *S. distigmaticus* | Brown-off date | T*_gmax_* | 7 | 0.0306 | 0.8773 | 26 | 10 |
| *S. distigmaticus* | Brown-off date | T*_gmax_* | 6 | 0.1288 | 0.5136 | 26 | 45 |
| *S. distigmaticus* | Brown-off date | T*_gmean_* | 9 | 0.5524 | 0.0023 | 26 | 99 |
| *S. distigmaticus* | Brown-off date | T*_gmean_* | 8 | 0.5098 | 0.0056 | 26 | 99 |
| *S. distigmaticus* | Brown-off date | T*_gmean_* | 7 | 0.2610 | 0.1797 | 26 | 80 |
| *S. distigmaticus* | Brown-off date | T*_gmean_* | 6 | 0.4775 | 0.0102 | 26 | 95 |
| *S. distigmaticus* | Brown-off date | T*_min_* | 9 | 0.5581 | 0.0020 | 26 | 99 |
| *S. distigmaticus* | Brown-off date | T*_min_* | 8 | 0.5630 | 0.0018 | 26 | 99 |
| *S. distigmaticus* | Brown-off date | T*_min_* | 7 | 0.3607 | 0.0594 | 26 | 90 |
| *S. distigmaticus* | Brown-off date | T*_min_* | 6 | 0.4971 | 0.0071 | 26 | 99 |
| *S. distigmaticus* | Brown-off date | T*_max_* | 9 | 0.2837 | 0.1435 | 26 | 85 |
| *S. distigmaticus* | Brown-off date | T*_max_* | 8 | 0.4490 | 0.0165 | 26 | 95 |
| *S. distigmaticus* | Brown-off date | T*_max_* | 7 | 0.2101 | 0.2833 | 26 | 70 |
| *S. distigmaticus* | Brown-off date | T*_max_* | 6 | 0.1645 | 0.4029 | 26 | 55 |
| *S. distigmaticus* | Brown-off date | T*_mean_* | 9 | 0.5121 | 0.0053 | 26 | 99 |
| *S. distigmaticus* | Brown-off date | T*_mean_* | 8 | 0.5672 | 0.0016 | 26 | 99 |
| *S. distigmaticus* | Brown-off date | T*_mean_* | 7 | 0.3404 | 0.0763 | 26 | 90 |
| *S. distigmaticus* | Brown-off date | T*_mean_* | 6 | 0.3736 | 0.0502 | 26 | 90 |
| *S. distigmaticus* | Brown-off date | Pre | 9 | 0.3428 | 0.0741 | 26 | 90 |
| *S. distigmaticus* | Brown-off date | Pre | 8 | 0.2472 | 0.2047 | 26 | 75 |
| *S. distigmaticus* | Brown-off date | Pre | 7 | 0.1175 | 0.5514 | 26 | 40 |
| *S. distigmaticus* | Brown-off date | Pre | 6 | 0.1183 | 0.5489 | 26 | 45 |
| *S. distigmaticus* | Brown-off date | Ssd | 9 | -0.1028 | 0.6028 | 26 | 35 |
| *S. distigmaticus* | Brown-off date | Ssd | 8 | 0.1408 | 0.4747 | 26 | 50 |
| *S. distigmaticus* | Brown-off date | Ssd | 7 | -0.0199 | 0.9199 | 26 | 5 |
| *S. distigmaticus* | Brown-off date | Ssd | 6 | -0.0102 | 0.9588 | 26 | 1 |
| *S. distigmaticus* | Brown-off date | H*_min_* | 9 | -0.0547 | 0.7821 | 26 | 20 |
| *S. distigmaticus* | Brown-off date | H*_min_* | 8 | -0.3451 | 0.0721 | 26 | 90 |
| *S. distigmaticus* | Brown-off date | H*_min_* | 7 | -0.2791 | 0.1503 | 26 | 80 |
| *S. distigmaticus* | Brown-off date | H*_min_* | 6 | -0.3471 | 0.0704 | 26 | 90 |
| *S. distigmaticus* | Brown-off date | H*_mean_* | 9 | -0.1124 | 0.5689 | 26 | 40 |
| *S. distigmaticus* | Brown-off date | H*_mean_* | 8 | -0.3342 | 0.0822 | 26 | 90 |
| *S. distigmaticus* | Brown-off date | H*_mean_* | 7 | -0.3222 | 0.0945 | 26 | 90 |
| *S. distigmaticus* | Brown-off date | H*_mean_* | 6 | -0.3736 | 0.0502 | 26 | 90 |
| *S. distigmaticus* | Brown-off date | W*_mean_* | 9 | 0.2026 | 0.3011 | 26 | 65 |
| *S. distigmaticus* | Brown-off date | W*_mean_* | 8 | 0.1232 | 0.5323 | 26 | 45 |
| *S. distigmaticus* | Brown-off date | W*_mean_* | 7 | 0.0493 | 0.8031 | 26 | 15 |
| *S. distigmaticus* | Brown-off date | W*_mean_* | 6 | -0.1424 | 0.4699 | 26 | 50 |
| *S. distigmaticus* | Brown-off date | W*_max_* | 9 | -0.4735 | 0.0109 | 26 | 95 |
| *S. distigmaticus* | Brown-off date | W*_max_* | 8 | -0.6303 | 0.0003 | 26 | 99 |
| *S. distigmaticus* | Brown-off date | W*_max_* | 7 | -0.6702 | 0.0001 | 26 | 99 |
| *S. distigmaticus* | Brown-off date | W*_max_* | 6 | -0.6883 | 0.0001 | 26 | 99 |
| *S. distigmaticus* | Brown-off date | Eva | 9 | -0.7343 | 0.0000 | 26 | 99 |
| *S. distigmaticus* | Brown-off date | Eva | 8 | -0.5788 | 0.0013 | 26 | 99 |
| *S. distigmaticus* | Brown-off date | Eva | 7 | -0.6058 | 0.0006 | 26 | 99 |
| *S. distigmaticus* | Brown-off date | Eva | 6 | -0.6218 | 0.0004 | 26 | 99 |

**Supplementary Table 8** Stepwise regression coefficients of herb green-up dates and the selected climatic factors in optimum LP

| Species | Selected climate factor | regression coefficients | t-statistics | df | P-value | Standard error | Confidence interval  (%) |
| --- | --- | --- | --- | --- | --- | --- | --- |
| *E. nutans* | T*_gmean_* | -1.72 d/℃ | -3.26 | 25 | 0.0032 | 3.25 | 99 |
| *E. nutans* | T*_max_* | 1.22 d/℃ | 2.69 | 25 | 0.0124 | 3.25 | 95 |
| *K. pygmaea* | H*_mean_* | -0.51 d/% | -3.00 | 26 | 0.0059 | 3.81 | 99 |
| *P. asiatica* | T*_gmean_* | -2.49 d/℃ | -4.03 | 27 | 0.0007 | 2.51 | 99 |
| *P. asiatica* | Ssd | 0.09 d/h | 2.57 | 27 | 0.0186 | 2.51 | 95 |
| *P. tenuiflora* | T*_gmean_* | -1.20 d/℃ | -2.31 | 25 | 0.0294 | 3.42 | 95 |
| *P. tenuiflora* | Eva | 0.09 d/10mm | 2.40 | 25 | 0.0241 | 3.42 | 95 |
| *S. distigmaticus* | T*_min_* | 1.52 d/℃ | 2.51 | 25 | 0.0191 | 3.15 | 95 |
| *S. distigmaticus* | H*_mean_* | 0.62 d/% | -4.44 | 25 | 0.0002 | 3.15 | 99 |

**Supplementary Table 9** Stepwise regression coefficients of herb brown-off dates and the selected climate factors in optimum LP

| Species | Selected climate factor | regression coefficients | t-statistics | df | P-value | Standard error | Confidence interval  (%) |
| --- | --- | --- | --- | --- | --- | --- | --- |
| *E. nutans* | T*_max_* | 5.10 d/℃ | -4.08 | 26 | 0.0004 | 4.58 | 99 |
| *K. pygmaea* | Eva | -0.04 d/10mm | -3.67 | 26 | 0.0011 | 4.81 | 99 |
| *P. asiatica* | T*_gmin_* | 5.35 d/℃ | 6.60 | 26 | 0.0000 | 3.18 | 99 |
| *P. asiatica* | W*_mean_* | 10.70 d/m/s | 2.11 | 26 | 0.0489 | 3.18 | 95 |
| *P. asiatica* | W*_max_* | -6.26 d/m/s | -3.01 | 26 | 0.0074 | 3.18 | 99 |
| *P. tenuiflora* | T*_gmin_* | 3.64 d/℃ | 2.59 | 24 | 0.0160 | 6.09 | 95 |
| *P. tenuiflora* | W*_mean_* | 18.10 d/m/s | 3.49 | 24 | 0.0019 | 6.09 | 99 |
| *P. tenuiflora* | W*_max_* | -8.97 d/m/s | -2.60 | 24 | 0.0157 | 6.09 | 95 |
| *S.* *distigmaticus* | T*_gmin_* | 9.68 d/℃ | 8.18 | 25 | 0.0000 | 7.80 | 99 |
| *S.* *distigmaticus* | H*_mean_* | -2.21 d/% | -2.20 | 25 | 0.0377 | 7.80 | 95 |
